# End‐to‐End Pierced Carbon Nanosheets with Meso‐Holes

**DOI:** 10.1002/advs.202409546

**Published:** 2024-11-25

**Authors:** Minjun Kim, Hiroki Nara, Yusuke Asakura, Takashi Hamada, Peng Yan, Jacob Earnshaw, Meng An, Miharu Eguchi, Yusuke Yamauchi

**Affiliations:** ^1^ Australian Institute for Bioengineering and Nanotechnology (AIBN) The University of Queensland Queensland 4072 Australia; ^2^ Faculty of Science and Engineering and Waseda Research Institute for Science and Engineering Waseda University 3‐4‐1 Okubo Shinjuku Tokyo 169–8555 Japan; ^3^ Department of Materials Process Engineering Graduate School of Engineering Nagoya University Nagoya Aichi 464–8603 Japan; ^4^ College of Mechanical and Electrical Engineering Shaanxi University of Science and Technology Xi'an 710021 China

**Keywords:** 2D carbons, aluminosilicates, meso‐holey carbon nanosheets, micelles, open *Z*‐axis passage

## Abstract

The remarkable properties of 2D nanomaterials are well known. However, their high interfacial adhesion energy often leads to restacking issues, limiting their potential in various applications. A strategic synthetic approach is presented to overcome this challenge. Specifically, the study first demonstrates the use of layered aluminosilicate as a sacrificial 2D template to allow the growth of highly ordered meso‐holey polymeric layers, which can be subsequently exfoliated upon the removal of aluminosilicate and thermally converted to perpendicularly open meso‐holey carbon (POMC). On the other hand, perpendicularly blocked meso‐holey carbon (PBMC) is obtained with non‐sacrificial 2D template of graphene oxide. When both POMC and PBMC are evaluated by *operando* hydrodynamic electrochemical impedance spectroscopy and transmission line model analysis for electrochemical reduction of oxygen, POMC achieves a remarkable improvement of charge transfer and mass transfer by up to 4.1 and 7.9 times, respectively, as compared to PBMC. This study therefore highlights the importance of perpendicularly open 2D nanoarchitectures in circumventing the restacking effect, offering valuable insights for leveraging 2D nanomaterials with open meso‐holes in various applications.

## Graphical abstract

The exfoliation‐induced meso‐hole opening approach is first demonstrated to synthesize 2D carbon nanosheets with end‐to‐end pierced meso‐holes using the sacrificial 2D aluminosilicate. With open meso‐holes significantly improving mass transfer by up to 7.9 times, the crucial role of mass transfer effects over charge transfer effects in electrochemical reaction kinetics is demonstrated, highlighting the importance of open 2D nanoarchitectures.

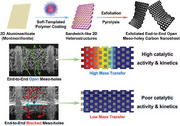

## 1 Introduction

2D nanomaterials are renowned for their unique physical, chemical, electronic, and optical properties which are mainly attributed to the thin layer structure consisting of strong in‐plane covalent bonds. Especially, the anisotropic nature of 2D nanomaterials results in a large surface area and potentially exposes more chemical sites for adsorption or catalytic activity.^[^ ^1^ ^]^ Graphene, for example, is an atomically thin 2D nanomaterial consisting of *sp* ^2^‐hybridized carbon atoms and its anisotropic 2D structure exhibits an extremely large theoretical surface area (≈2650 m^2^ g^−1^).^[^ ^2^ ^]^ Although such a large theoretical surface area of graphene has attracted significant interest in the field of materials science, their high interfacial adhesion energy causes a severe restacking between individual graphenes. As a result of restacking, the actual surface area measured through physical or electrochemical adsorption behavior is significantly lower (≈50 m^2^ g^−1^) than the theoretical value, therefore limiting the potential of graphene in various applications such as energy storage/conversion, sensing, chemical/drug delivery, purification, catalysis, *etc*.^[^ ^3^, ^4^, ^5^ ^]^ Particularly, their restacking creates potential problems associated with hampered mass transfer and partial embedment of the active sites on the basal plane.^[^ ^6^ ^]^ The issue of restacking is common in most 2D nanomaterials, and there has been a notable effort to overcome the adverse effect of structural restacking through a number of different approaches.^[^ ^7^, ^8^, ^9^, ^10^ ^]^ Among them, the creation of highly ordered nanopores or holes of tunable size in the 2D nanomaterials is a simple and effective measure to overcome the severe restacking effect.^[^ ^11^ ^]^ For instance, surfactants or block copolymers were widely implemented as soft‐templates to create nanoporous layers on the surface of 2D template materials, therefore, forming layer‐by‐layer 2D porous heterostructures. Such sandwich‐like porous heterostructures generally possess much larger accessible surface area and facilitates significantly improved mass transfer of chemical species.^[^ ^12^, ^13^, ^14^, ^15^ ^]^ Despite certain structural benefits, the sandwich‐like 2D heterostructures fail to utilize the *z*‐axis passages due to the presence of the 2D template materials acting as the physical barrier between the nanoporous layers, restricting the mass transfer. Alternatively, the micellization of block copolymers and polymerization of carbon precursors can be induced at the interface between immiscible phases to achieve the anisotropic growth of 2D porous polymer, which can be successfully converted to porous carbon nanosheets upon thermal annealing.^[^ ^16^, ^17^ ^]^ Though this method allows the open *z*‐axis passages of the carbon nanosheets, it potentially suffers from limited scalability and disordered mesopores in terms of arrangement and size because the formation of highly uniform micelles at the interface may not be easy.

Herein, this work presents end‐to‐end pierced meso‐holey carbon nanosheets with a high ordering in pore arrangement and size. To achieve this, meso‐holey polydopamine (mPDA) layers are formed on a type of layered aluminosilicate, known as montmorillonite (MMT), initially forming a typical sandwich‐like 2D heterostructure (MMT@mPDA). The removal of the sacrificial 2D template (MMT) causes an unusual exfoliation of mPDA layers, therefore, resulting in 2D nanosheets with highly ordered open meso‐holes. The exfoliated open meso‐holey 2D nanosheets are subsequently subjected to direct‐carbonization to yield perpendicularly open meso‐holey carbon (POMC). Finite element simulations of the OH^−^ diffusion demonstrate that the open meso‐holes of POMC allows enormously greater rate of diffusion as compared to perpendicularly blocked meso‐holey carbon (PBMC) derived from graphene oxide (GO) coated with mPDA (GO@mPDA). Moreover, *operando* hydrodynamic electrochemical impedance spectroscopy (EIS) and transmission line model (TLM) analysis further reveal that POMC facilitates extremely high mass transfer by achieving up to 4.1‐ and 7.9‐times lower resistance for charge transfer and mass transfer, respectively, as compared to PBMC.

## 2 Results and Discussion

**Figure 1:**
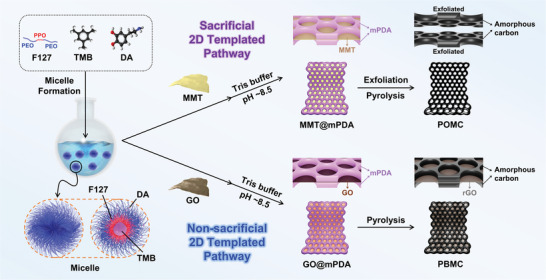
Schematic illustration of the synthesis processes to obtain PBMC and POMC.

To investigate the effect of perpendicularly open meso‐holes of 2D carbon nanosheets, POMC and PBMC were synthesized as described in **Figure** **1**. Typically, the combination of PEO_100_‐PPO_65_‐PEO_100_ triblock copolymer (F127) and 1,3,5‐trimethylbenzene (TMB) was implemented to induce the formation of micelles. In the process of micellization, TMB acts as a hydrophobic core of the micelle and attracts the hydrophobic PPO chains of F127 while PEO chains interact with the aqueous environment. Subsequently, dopamine (DA) monomers and 2D templates (MMT for POMC and GO for PBMC) were introduced to the system. Specifically, the PEO groups of F127, catechol and ethylamine groups of DA, and water molecules in the system interacted with each other by hydrogen bonds, forming stable F127/TMB/DA micelle composites to induce the formation of mesopores upon polymerization.^[^ ^18^, ^19^ ^]^ It is important to note that MMT acts as a “sacrificial” 2D template while GO acts as a “non‐sacrificial” 2D template to allow the growth of meso‐holey polymeric layers and form sandwich‐like 2D heterostructure of MMT@mPDA and GO@mPDA, respectively. With F127/TMB micelles, the channel‐like growth of the mPDA layer is induced to form a meso‐holey structure on the respective 2D template.^[^ ^20^, ^21^, ^22^ ^]^

**Figure 2:**
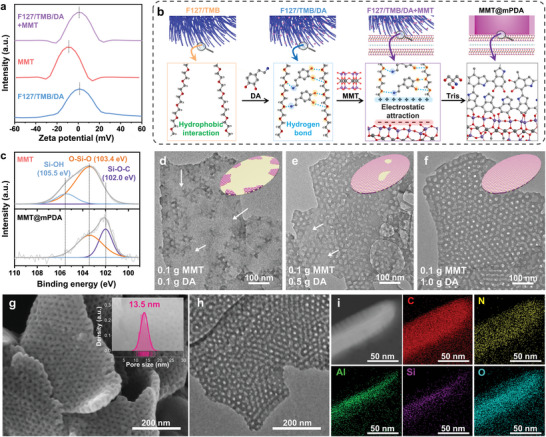
a) Zeta potential distribution curves. b) Schematic illustration of the interaction between F127/TMB micelle, DA and MMT based on the zeta potential analysis. c) High‐resolution XPS spectra for Si 2*p* of MMT and MMT@mPDA. TEM images of MMT (0.1 g) coated by d) 0.1 g DA, e) 0.5 g DA, and f) 1.0 g DA, respectively. g) SEM, h) TEM, and i) cross‐sectional EDS images of MMT@mPDA. Note: the inset of (g) represents the pore size distribution of MMT@mPDA obtained by “ImageJ” software.

To gain a better understanding of the type of chemical interactions between F127/TMB/DA micelle composites and MMT, the change in zeta potential during the synthesis of MMT@mPDA was evaluated. The zeta potential of F127/TMB/DA micelle composites is detected at +1.63 mV while MMT itself presents a negative zeta potential of −9.06 mV (**Figure** **2**a). The negative zeta potential of MMT can be attributed to the permanent negative charge on its basal plane,^[^ ^23^ ^]^ thus making it able to electrostatically interact with F127/TMB/DA micelle composites that have the oppositely charged surface (Figure 2a). Upon mixing of MMT and F127/TMB/DA micelle composites, the overall zeta potential shifts to +1.05 mV, indicating that the negatively charged surface of MMT is effectively covered by the positively charged F127/TMB/DA micelle composites (Figure 2a). Consequently, the formation of mPDA occurs on the surface of MMT to give rise to MMT@mPDA. The suggested chemical interactions in each stage of the synthesis of MMT@mPDA are described in Figure 2b.

X‐ray photoelectron spectroscopy (XPS) spectra of MMT and MMT@mPDA indicate that the XPS peak intensities of elements originating from MMT (Al, Si, and Mg) are significantly reduced while the peak intensities for C and N originating from mPDA increase (Figure S1, Supporting Information). Such dramatic change in the surface chemical environment indicates that the polymeric coating is successfully applied to MMT. High‐resolution XPS spectra for Si 2*p* reveal that the mPDA coating causes a significant reduction of the peak for Si─OH bonds (105.5 eV) but the clear appearance of another peak for Si─O─C bonds (102.0 eV) (Figure 2c).^[^ ^24^ ^]^ Based on the XPS analysis, it can be inferred that the exposed silanol groups (Si─OH) of MMT and catechol (─C─OH) groups of DA potentially undergo condensation reactions to a form of Si─O─C bonds in MMT@mPDA, therefore achieving a homogeneous mPDA coating (Figure S2, Supporting Information). The transmission electron microscope (TEM) images demonstrate that the initial mPDA coating takes place from the edges of MMT and the polymeric chain extends toward the center of the basal plane, eventually achieving a full mPDA coating on the basal plane of MMT (Figure 2d–f).

Highly ordered and uniform meso‐holes with the mean diameter of 13.5 nm are clearly observed from the scanning electron microscope (SEM) image of MMT@mPDA (Figure 2g). From the transmitted image of MMT@mPDA, the meso‐holes from the top and bottom mPDA layers appear overlaid on each other, indicating that it demonstrates sandwich‐like 2D heterostructure (Figure 2h). In addition, cross‐sectional dark‐field scanning TEM (DF‐STEM) image and energy‐dispersive X‐ray spectroscopy (EDS) mappings of MMT@mPDA further confirm the sandwich‐like 2D heterostructure with Si and Al atoms originating from MMT are concentrated in the center while C and N atoms originating from mPDA are homogeneously distributed (Figure 2i).

**Figure 3:**
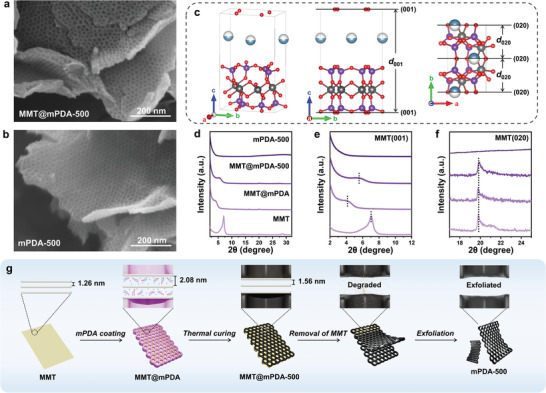
SEM images of a) MMT@mPDA‐500 and b) mPDA‐500. c) Schematic descriptions of MMT unit cells. d) Wide‐range X‐ray diffractograms of all samples (MMT, MMT@mPDA, MMT@mPDA‐500, and mPDA‐500). Narrow‐range X‐ray diffractograms of all samples showing e) MMT(001) and f) MMT(020). g) Schematic illustration of the proposed exfoliation mechanism.

To successfully obtain perpendicularly open meso‐holes, the sacrificial 2D template MMT was removed while maintaining mPDA layers. Though in situ generated HF solution (the mixture of LiF and HCl commonly used to etch Al from Ti_3_AlC_2_ MAX phase)^[^ ^25^ ^]^ can effectively etch MMT, it also causes undesired dissolution of the mPDA layer due to the highly acidic and corrosive nature. Therefore, before the etching, we first applied a thermal curing of the mPDA layer to induce further cross‐linking and achieve additional chemical stability. According to the SEM images, the meso‐holey structure of MMT@mPDA is well‐maintained after the thermal curing at 500 °C (**Figure** **3**a). Additionally, SEM and TEM images of the cured sample (MMT@mPDA‐500) demonstrate a partial exposure of MMT at which the partial thermal decomposition of mPDA layer seems to have occurred (Figure S3, Supporting Information). Interestingly, the HF etching causes the two mPDA‐500 layers to be exfoliated into individual end‐to‐end open meso‐holey 2D nanosheets upon the removal of MMT (Figure 3b).

The exfoliation mechanism of MMT@mPDA‐500 to individual mPDA‐500 layers upon the removal of MMT was further investigated by X‐ray diffraction (XRD). Based on the unit cell structure of MMT in Figure 3c, MMT(001) is the basal plane with the out‐of‐plane direction. As the interlayer spacing of the basal plane (*d*_001_) is affected by the electrostatic attraction between the permanent negative surface of the basal planes and intercalating cations (e.g., Mg^2+^ according to XPS analysis, Figure S1, Supporting Information), the *d*_001_ can be changed by the addition or removal of intercalating species. Indeed, there is a notable shift of diffraction peak at 7.03°, corresponding to MMT(001), to 4.24° in MMT@mPDA, indicating the increased *d*_001_ from 1.26 to 2.08 nm after the mPDA coating (Figure. 3d,e). Such an increase in the value of *d*_001_ can be attributed to the presence of residual F127 intercalating and expanding the basal planes of MMT during the synthesis of MMT@mPDA.^[^ ^26^ ^]^ After the thermal curing at 500 °C, the MMT(001) peak slightly increases to 5.64° due to the thermal decomposition of intercalated F127 and dehydration, thus decreasing the *d*_001_ to 1.56 nm (Figure. 3d,e).^[^ ^27^ ^]^

While there is a prominent shift of the diffraction peak of the basal plane of MMT after each treatment, it must be noted that the diffraction peak for MMT(020) remains unshifted in MMT, MMT@mPDA and MMT@mPDA‐500 (Figure 3f). As the in‐plane covalent bonds of MMT are formed at specific bond lengths and angles, the change in the interlayer spacing of MMT(020) (*d*_020_) can be induced by the bond breaking and/or formation (Figure 3c). However, as MMT, MMT@mPDA and MMT@mPDA‐500 present the unshifted diffraction peak for MMT(020), it can be inferred that each treatment preserves in‐plane covalent bonds of MMT. Upon the etching of MMT, the breaking of in‐plane covalent bonds of MMT occurs as demonstrated by the complete disappearance of diffraction peak for MMT(020) in mPDA‐500 (Figure 3d,f). The effective removal of MMT from MMT@mPDA‐500 was achieved as the exposed and expanded basal planes [i.e., MMT(001)] offer facile access for the etchant to contact and enter the interlayer space, promoting the exfoliation of mPDA‐500 layers (Figure 3g).

**Figure 4:**
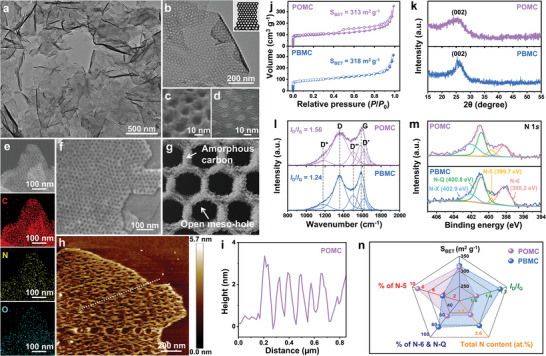
a,b) TEM, c) SE‐STEM, d) high‐resolution TEM, e) EDS, and f) SEM images of POMC. g) Schematic illustration of POMC. h) AFM images and i) corresponding height profiles of POMC. j) Nitrogen adsorption‐desorption isotherms of PBMC and POMC. k) X‐ray diffraction spectra of PBMC and POMC. l) Raman spectra of PBMC and POMC. m) High‐resolution XPS spectra for N 1*s* of PBMC and POMC. n) Radar chart of PBMC and POMC.

The exfoliated mPDA‐500 is finally subjected to thermal annealing at 900 °C to induce carbonization and obtain POMC (Figure S4a,b, Supporting Information). The TEM images of POMC demonstrate that the meso‐holes are well‐reserved even after the high‐temperature pyrolysis at 900 °C (**Figure** **4**a,b and Figure S4c, Supporting Information). From secondary electron STEM (SE‐STEM) and high‐resolution TEM images, smaller nanopores are observed on the pore walls next to the ordered mesopores (Figure 4c,d and Figure S4d, Supporting Information). Because SE‐STEM and DF‐STEM images offer surface information and transmitted internal information, respectively, they can effectively verify the openness of the meso‐holes in POMC. As expected, the meso‐holes of POMC from both SE‐STEM and DF‐STEM images match well with each other, confirming the perpendicular openness of meso‐holes (Figure S4e,f, Supporting Information). Moreover, EDS mappings show that the carbon atoms are concentrated along the pore walls of meso‐holes in the DF‐STEM image, thus indicating that the meso‐holes are perpendicularly open without being blocked by other elements (Figure 4e). The exfoliated monolayer POMC is also demonstrated by the SEM image (Figure 4f) and its end‐to‐end pierced meso‐holes are described in Figure 4g.

On the other hand, GO@mPDA was successfully obtained with GO as the non‐sacrificial template (Figure S5a–d, Supporting Information). Upon thermal annealing at 900 °C, GO@mPDA was successfully converted to PBMC with well‐preserved meso‐holes demonstrated by the SEM and TEM images (Figure S5e,f, Supporting Information). It is also noted that reduced GO (rGO) is present at the center of the sandwich‐like 2D heterostructure, thus blocking the perpendicular passage of meso‐holes (Figure S6a–f, Supporting Information). According to AFM analysis, the height profile of POMC indicates the perpendicular openness as the height fluctuation due to porous contour reaches the substrate while PBMC exhibits a significantly reduced height fluctuation, suggesting that it is perpendicularly blocked (Figure 4h,i and Figure S6g,h, Supporting Information).

According to nitrogen adsorption‐desorption isotherms, though POMC and PBMC have similar specific surface area (*S*_BET_) values of ≈315 m^2^ g^−1^ due to highly uniform meso‐holes of similar sizes on the surface, their isotherm patterns are quite different from each other (Figure 4j). Specifically, the nitrogen adsorption‐desorption isotherm of POMC involves a clear hysteresis due to the different nitrogen adsorption and desorption mechanisms in the perpendicularly open meso‐holes as described in Figure S7a (Supporting Information). On the other hand, such hysteresis is not clearly observed from the isotherm of PBMC potentially due to the presence of rGO also serving as the nucleation site for condensation, allowing the rate of nitrogen adsorption and desorption to be similar (Figure S7b, Supporting Information).

Carbon structures of POMC and PBMC were then investigated with various characterization methods. XRD analysis further verifies the presence of rGO in PBMC as it presents a sharper diffraction peak at ≈26° [corresponding to (002) lattice plane of graphite] than POMC (Figure 4k). Due to the presence of amorphous carbon derived from the polymeric layers, the diffraction peak for (002) is broadened for both POMC and PBMC. Amorphous carbon structures of POMC and PBMC were further studied by Raman spectroscopy. Their Raman spectra are first deconvoluted to D^*^ (1180 cm^−1^), D (1357 cm^−1^), D′′ (1501 cm^−1^), G (1587 cm^−1^), and D′ (1618 cm^−1^) bands of carbon lattice (Figure 4l). Specifically, D^*^, D, D′′, and D′ bands arise from the defects and disorders in the carbon structure while G band origniates from the in‐plane vibrational mode of *sp* ^2^‐hybridized carbon atoms, therefore, the intensity ratio of D and G bands (*I*_D_/*I*_G_) indicates the level of graphitization of the carbon materials.^[^ ^28^, ^29^, ^30^ ^]^ Based on the deconvoluted Raman spectra, *I*_D_/*I*_G_ value of PBMC (1.24) is calculated to be lower than that of POMC (1.56), indicating that it has a greater level of *sp* ^2^‐hybridized carbon atoms and confirming the existence of rGO predominantly consisting of *sp* ^2^‐hybridized carbons in PBMC.

To reveal the chemical compositions and bonds in the carbon structure, XPS analysis was subsequently conducted. From the survey XPS spectra, both POMC and PBMC are composed of carbon, nitrogen, and oxygen atoms with highly similar atomic compositions (Figure S8a, Supporting Information). High‐resolution XPS for C 1*s* spectra indicate that POMC and PBMC are heteroatom‐doped with nitrogen atoms because there is a high level of C─N bonds in their carbon structure (Figure S8b,c, Supporting Information). To verify the nature of heteroatom doping, the deconvoluted of N 1*s* spectra of POMC and PBMC are analyzed (Figure 4m). The deconvoluted N 1*s* spectra of PBMC typically show a much higher level of pyridinic nitrogen (N‐6) and graphitic nitrogen (N‐Q) as compared to that of POMC, potentially due to the heteroatom‐doping of nitrogen to carbon matrix without forming much defective sites (Figure S8d, Supporting Information). On the other hand, POMC possesses more than twice the content of pyrrolic nitrogen (N‐5) than PBMC, indicating that it involves high level of defective sites in the carbon structure (Figure S8d, Supporting Information). Based on the above analysis, it is confirmed that both POMC and PBMC exhibit very similar morphological characteristics (meso‐hole size and specific surface area). Nonetheless, their carbon structures are quite different from each other with POMC having more defective sites than PBMC despite having similar overall nitrogen content. This also possibly contributes to the difference in their level of graphitization (Figure 4n).

To demonstrate the versatility of this synthetic approach for an additional heteroatom‐doping, sulfur‐containing DA‐like monomer (S‐DA) was implemented for the polymerization (More details on the synthesis of S‐DA, MMT@mPDA‐S and S‐POMC can be found in Supporting Information). Even with the incorporation of S‐DA, the resulting sandwich‐like 2D heterostructure (MMT in between the polymeric layers involving both nitrogen and sulfur atoms, MMT@mPDA‐S) maintains highly uniform mesopores (Figure S9, Supporting Information). The final carbon materials (S‐POMC) obtained via the same exfoliation‐induced approach demonstrates clear meso‐holes with dual heteroatoms of nitrogen and sulfur (Figure S10, Supporting Information). These results demonstrate a great potential for the exfoliation‐induced meso‐hole openning approach for heteroatom engineering for various applications.

**Figure 5:**
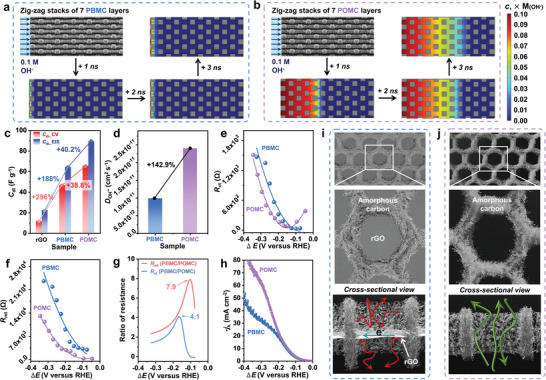
Concentration profile of OH^−^ through the seven‐stacks of a) PBMC and b) POMC in zig‐zag arrangement obtained by finite element simulation along the time span of 1 to 6 ns. c) *C*_dl__CV (dynamic condition) and *C*_dl__EIS (static condition) of rGO, PBMC and POMC. e) *R*_ct_ and f) *R*_mt_ of PBMC and POMC obtained from *operando* hydrodynamic EIS measured at the potentials marked in Figure S9 (Supporting Information). g) Ratio of resistance for *R*_ct_ and *R*_mt_ between PBMC and POMC. h) *j*_k_ of PBMC and POMC. Schematic description of i) open meso‐holes and amorphous carbon matrix of POMC and j) blocked meso‐holes and amorphous carbon matrix of PBMC embedding rGO. Note: In *operando* hydrodynamic EIS analysis, the effect of *E*_onset_ is compensated by subtracting *E*_onset_ from *E*_applied_ (*i.e*., *ΔE* = *E*_applied_ *– E*_onset_) and the resistance is normalized by the electrode thickness of PBMC and POMC.

With an in‐depth understanding of POMC and PBMC from morphological and structural analyses, the effect of the perpendicularly open or blocked meso‐holes on the restacking and mass transfer is theoretically invesitgated by finite element simulations. For the simulation, the simplified 2D models consisting of seven stacks of POMC and PBMC in zig‐zag arrangement are adopted as shown in Figure S11 (Supporting Information) (More details on the simulation can be found in Supporting Information). To the simplified models, the flow of 0.1 M OH^−^ is applied from the left side of the stack with a voltage bias of −1.2 V along the direction of the decreasing concentration to achieve the concentration profile of OH^−^ with respect to time factor in the 2D models. A striking difference in the diffusion efficiency of OH^−^ between POMC and PBMC is demonstrated even in the very short time span of 6 ns with a severely decreased mass transfer of OH^−^ in PBMC due to the presence of rGO blocking the meso‐holes (**Figure** **5**a). On the contrary, the open meso‐holes of POMC endow highly continuous diffusion passages for efficient mass transfer of OH^−^, further signifying the importance of end‐to‐end pierced meso‐holes on 2D carbon nanosheets in alleviating the restacking effect for potential applications (Figure 5b).

For the initial electrochemical analysis by double‐layer capacitance, rGO was also considered to serve as 2D carbon nanosheets with no meso‐holes to further highlight the presence of meso‐holes in blocked or open state (Figure S12, Supporting Information). To identify the electrochemical relevance of the end‐to‐end pierced meso‐holes of 2D carbon nanosheets, electrochemically active and wettable surface areas are subsequently evaluated by double layer capacitance derived from cyclic voltammetry (*C*_dl__CV) and EIS (*C*_dl__EIS), respectively (Figure S13, Supporting Information). Specifically, rGO is only capable of achieving *C*_dl__CV and *C*_dl__EIS of 11.8 and 22.4 F g^−1^, respectively. As the carbon nanosheet incorporates blocked meso‐holes in the sandwich‐like 2D heterostructure of PBMC, both *C*_dl__CV and *C*_dl__EIS increase by 296% and 188% to 46.9 and 64.5 F g^−1^, respectively, from that of rGO (Figure 5c). This indicates that there is an obvious structural benefit even in the sandwich‐like 2D heterostructure with blocked meso‐holey layers to alleviate the restacking effect and increase the electrochemically accessible and wettable surface areas. Moreover, POMC achieves even further increase from the *C*_dl__CV and *C*_dl__EIS of PBMC by 38.8% and 40.2%, respectively, despite both having similar *S*_BET_ values (Figure 5c). This clearly demonstrates that the open meso‐holes facilitate more efficient utilization of the surface area in electrochemistry as compared to the blocked meso‐holes. Next, electrocatalytic activity for the reduction of oxygen is evaluated for rGO, PBMC, and POMC with forced convection to gain further understanding on the use of their surface area for electrocatalytic reactions. Based on the linear sweep voltammetry (LSV) curves at 1600 rpm, the trend of catalytic activities of rGO, PBMC, and POMC for the electrochemical reduction of oxygen matches well with that *C*_dl__CV and *C*_dl__EIS values, therefore, indicating the importance of structural openness in facilitating efficient electrochemical and electrocatalytic involvement of surface area of carbon nanosheets (Figure S14, Supporting Information). In the case of PBMC obtained by the same procedure as POMC (denoted as PBMC‐HF) involving thermal curing and HF treatment, it demonstrates improved electrochemical properties over rGO, but does not surpass that of PBMC (Figures S13 and S14, Supporting Information). As both rGO and PBMC‐HF present the poorest electrochemically accessible surface area and electrocatalytic activity, the following *operando* hydrodynamic EIS analysis was conducted with PBMC and POMC to focus on the significance of blocked or open state of meso‐holes (Figure S14, Supporting Information).

The impact of open or blocked meso‐holes of carbon nanosheet is subsequently studied by diffusion coefficient of OH^−^ (*D*_OH_^−^) calculated from the EIS. Specifcially, POMC significantly outperforms that of PBMC by 142.9%, indicating that the open meso‐holes are crucial for the improved mass transfer process (Figure 5d and Figure S15, Supporting Information). Additionally, *operando* hydrodynamic EIS analysis was performed for PBMC and POMC to gain more understanding on the mass transfer and charge transfer associated with the electrochemical reduction of oxygen. Typically, potential‐dependant charge transfer resistance (*R*_ct_) and mass transport resistance (*R*_mt_) for the electrochemical reduction of oxygen was calculated from the TLM and normalized by the electrode thickness (Figures S16 and S17, Supporting Information). When *ΔE* is close to 0, POMC presents significantly greater *R*_ct_ values than PBMC (Figure 5e). The low initial *R*_ct_ values of PBMC can be attributed to the high level of *sp* ^2^‐hybridized carbons in rGO, facilitating more facile charge transfer at the active sites at low overpotential.^[^ ^30^ ^]^ On the contrary, POMC is predominantly composed of the amorphous carbon, therefore exerting a greater resistance to charge transfer at the active sites. At greater overpotentials, *R*_ct_ for both POMC and PBMC increase steadily due to the increasingly more limited supply of O_2_ to the active sites while the current supply increases. Though both POMC and PBMC present increasing trend of *R*_ct_ at greater overpotential (i.e., lower *ΔE*), POMC generates consistently lower *R*_ct_ values than that of PBMC primarily due to improved diffusion of O_2_ through the open meso‐holes to facilitate enhanced O_2_ supply to the active sites.

Under the forced hydrodynamic condition with the rotating disk electrode (RDE), the porous electrode extends the diffusion layer to its interconnected porous network while the nonporous electrode limits the diffusion layer to its exposed surface (Figure S18, Supporting Information). As shown in Figure 5f, when *ΔE* is close to 0, both PBMC and POMC generate low initial *R*_mt_ because their electrochemically accessible surface is saturated with residing O_2_, thus allowing a readily supply of O_2_ to the active sites. As the overpotential increases, the residing O_2_ in the electrode is continuously consumed by reduction, and subsequent O_2_ mass transfer becomes increasingly more dependent on the nanoarchitecture of electrode materials. Specifically, at greater overpotential, the region of O_2_ depletion expands in the electrode, and the electrochemical reduction process becomes increasingly dictated by the diffusion of O_2_.^[^ ^31^ ^]^ Consequently, the thickness of the diffusion layer within the electrode decreases, leaving a substantial portion of the surface electrochemically inactive, therefore increasing the *R*_mt_ of the electrode. In contrast to the blocked meso‐holes of PBMC, the open meso‐holes of POMC help facilitate highly efficient diffusion of O_2_ in the electrode, thus reducing the electrochemically inactive portion of the electrode (i.e., increasing the thickness of the diffusion layer in the electrode). Such structural openness of POMC electrode, therefore, leads to significantly lower *R*_ct_ and *R*_mt_ by up to 4.1 and 7.9 times, respectively, in comparison to PBMC electrode (Figure 5g). Next, the effect of *R*_ct_ and *R*_mt_ are correlated to the kinetic current densities (*j*_k_) of electrochemical reduction of oxygen. When *ΔE* is close to 0, high *R*_ct_ of POMC tends to offset its low *R*_mt_, and vice versa for PBMC (Figure 5h). As a result, POMC and PBMC generate similar *j*_k_ values in the initial stage. However, both *R*_ct_ and *R*_mt_ of POMC become significantly lower than that of PBMC as *ΔE* decreases, resulting in a notable increase in *j*_k_ values to outperform PBMC at respective *ΔE*. The observed effects of open and blocked meso‐holes on mass and charge transfer are described in Figure 5i,j. The *operando* hydrodynamic EIS analysis therefore confirms a promising improvement of mass transfer achieved by the open meso‐holes as compared to the blocked meso‐holes arrayed on the 2D carbon nanosheets of POMC and PBMC, respectively, evidencing the highlighted importance of open meso‐holes of the 2D porous carbon nanosheets in various applications requiring efficient mass transfer. POMC also demonstrates high electrochemcial stability with no visible change in meso‐holes after 10 000 cycles of CV, advocating its high versatility for various electrochemical applications (Figure S19, Supporting Information).

## 3 Conclusion

Extending from the well‐known use of silica or aluminosilicate as hard‐templates as well as polymeric soft‐templates for pore generation, this work successfully demonstrates the mixed use of a sacrificial 2D template (layered aluminosilicate, MMT) and a micellar soft‐template to obtain end‐to‐end open meso‐holey carbon nanosheets via an exfoliation‐induced meso‐hole opening approach.^[^ ^32^, ^33^, ^34^, ^35^ ^]^ The key feature of this strategy lies in the use of MMT as a sacrificial 2D template for the growth of meso‐holey polymeric layers (mPDA), and manipulation of its interlayer space for the effective removal by in situ generated HF. As a comparison sample, 2D carbon nanosheets with perpendicularly blocked meso‐holes are also synthesized using GO as a non‐sacrificial 2D template (PBMC). Particularly, the open meso‐holes of POMC are found to facilitate a significant improvement in electrochemical surface utilization (increase by ≈40%) and diffusion efficiency (increase by ≈143%) as compared to the blocked meso‐holes of PBMC. *Operando* hydrodynamic EIS and TLM analysis further demonstrates the structural advantages of POMC attributed to the open meso‐holes by achieving up to 4.1 and 7.9 times lower *R*_ct_ and *R*_mt_ than PBMC for the electrochemical reduction of oxygen. This work further advocates the importance of end‐to‐end open 2D nanoarchitecture in maximizing the use of surface area and mass transfer efficiency toward various applications that are limited by surface area and diffusion.

## Conflict of Interest

The authors declare no conflict of interest.

## Author Contributions

M.K. and Y.Y. designed the experiments, analyzed the data, and wrote the manuscript. M.K., T.H., and M.E. carried out the synthesis and basic characterization of materials. Y.A. performed the characterization of height and zeta potential profiles. P.Y. and M.A. carried out finite element simulations. M.K. and H.N. carried out the measurement of basic electrochemical activities and *operando* hydrodynamic EIS. H.N. performed the TLM analysis for mass transfer and charge transfer resistances. J.E. performed the measurement of Raman spectroscopy. All authors discussed the results and edited and commented on the manuscript.

## Supporting information



Supporting Information

## Data Availability

The data that support the findings of this study are available from the corresponding author upon reasonable request.

## References

[1] M. Zeng , Y. Xiao , J. Liu , K. Yang , L. Fu , Chem. Rev. 2018, 118, 6236. 29381058 10.1021/acs.chemrev.7b00633

[2] Y. Zhu , S. Murali , W. Cai , X. Li , J. W. Suk , J. R. Potts , R. S. Ruoff , Adv. Mater. 2010, 22, 3906. 20706983 10.1002/adma.201001068

[3] M. Pandey , K. Deshmukh , A. Raman , A. Asok , S. Appukuttan , G. R. Suman , Renewable Sustainable Energy Rev. 2024, 189, 114030.

[4] Y. Li , Y. Cui , M. Zhang , X. Li , R. Li , W. Si , Q. Sun , L. Yu , C. Huang , Nano Lett. 2022, 22, 2817. 35333055 10.1021/acs.nanolett.1c04976

[5] X. Liu , X. Wu , Y. Xing , Y. Zhang , X. Zhang , Q. Pu , M. Wu , J. X. Zhao , ACS Appl. Bio Mater. 2020, 3, 2577. 10.1021/acsabm.9b01108 35025390

[6] C. Tan , X. Cao , X.‐J. Wu , Q. He , J. Yang , X. Zhang , J. Chen , W. Zhao , S. Han , G.‐H. Nam , M. Sindoro , H. Zhang , Chem. Rev. 2017, 117, 6225. 28306244 10.1021/acs.chemrev.6b00558

[7] Z.‐S. Wu , Y. Sun , Y.‐Z. Tan , S. Yang , X. Feng , K. Müllen , J. Am. Chem. Soc. 2012, 134, 19532. 23148416 10.1021/ja308676h

[8] Y. Xia , T. S. Mathis , M.‐Q. Zhao , B. Anasori , A. Dang , Z. Zhou , H. Cho , Y. Gogotsi , S. Yang , Nature 2018, 557, 409. 29769673 10.1038/s41586-018-0109-z

[9] H. Chen , C.‐T. Hung , W. Zhang , L. Xu , P. Zhang , W. Li , Z. Zhao , D. Zhao , J. Am. Chem. Soc. 2023, 145, 27708. 38054893 10.1021/jacs.3c09927

[10] E. Pomerantseva , Y. Gogotsi , Nat. Energy 2017, 2, 17089.

[11] L. Peng , Z. Fang , J. Li , L. Wang , A. M. Bruck , Y. Zhu , Y. Zhang , K. J. Takeuchi , A. C. Marschilok , E. A. Stach , E. S. Takeuchi , G. Yu , ACS Nano 2018, 12, 820. 29261299 10.1021/acsnano.7b08186

[12] J. Wang , V. Malgras , Y. Sugahara , Y. Yamauchi , Nat. Commun. 2021, 12, 3563. 34117228 10.1038/s41467-021-23819-0 PMC8196154

[13] J. Wang , Z. Chang , B. Ding , T. Li , G. Yang , Z. Pang , T. Nakato , M. Eguchi , Y.‐M. Kang , J. Na , B. Y. Guan , Y. Yamauchi , Angew. Chem., Int. Ed. 2020, 59, 19570. 10.1002/anie.202007063 32652751

[14] X. Li , Q. Guan , Z. Zhuang , Y. Zhang , Y. Lin , J. Wang , C. Shen , H. Lin , Y. Wang , L. Zhan , L. Ling , ACS Nano 2023, 17, 1653. 10.1021/acsnano.2c11663 36607402

[15] Q. Li , X. Xu , J. Guo , J. P. Hill , H. Xu , L. Xiang , C. Li , Y. Yamauchi , Y. Mai , Angew. Chem., Int. Ed. 2021, 60, 26528. 10.1002/anie.202111823 34748252

[16] S. Kim , M. Ju , J. Lee , J. Hwang , J. Lee , J. Am. Chem. Soc. 2020, 142, 9250. 32053749 10.1021/jacs.0c00311

[17] W. Mai , Y. Zuo , X. Zhang , K. Leng , R. Liu , L. Chen , X. Lin , Y. Lin , R. Fu , D. Wu , Chem. Commun. 2019, 55, 10241. 10.1039/c9cc04664j 31393482

[18] J. Tang , J. Liu , C. Li , Y. Li , M. O. Tade , S. Dai , Y. Yamauchi , Angew. Chem., Int. Ed. 2015, 54, 588. 10.1002/anie.201407629 25393650

[19] P. Pan , T. Zhang , Q. Yue , A. A. Elzatahry , A. Alghamdi , X. Cheng , Y. Deng , Adv. Sci. 2020, 7, 2000443. 10.1002/advs.202000443 PMC7312473 32596127

[20] X. Yang , P. Lu , L. Yu , P. Pan , A. A. Elzatahry , A. Alghamdi , W. Luo , X. Cheng , Y. Deng , Adv. Funct. Mater. 2020, 30, 2002488.

[21] M. Kim , T. Park , C. Wang , J. Tang , H. Lim , M. S. A. Hossain , M. Konarova , J. W. Yi , J. Na , J. Kim , Y. Yamauchi , ACS Appl. Mater. Interfaces 2020, 12, 34065. 32686420 10.1021/acsami.0c07467

[22] M. Kim , K. L. Firestein , J. F. S. Fernando , X. Xu , H. Lim , D. V. Golberg , J. Na , J. Kim , H. Nara , J. Tang , Y. Yamauchi , Chem. Sci. 2022, 13, 10836. 36320690 10.1039/d2sc02726g PMC9491178

[23] L. Chen , Y. Zhao , H. Bai , Z. Ai , P. Chen , Y. Hu , S. Song , S. Komarneni , Langmuir 2020, 36, 10860. 32813528 10.1021/acs.langmuir.0c02073

[24] D. P. Narayanan , A. Gopalakrishnan , Z. Yaakob , S. Sugunan , B. N. Narayanan , Arab. J. Chem. 2020, 13, 318.

[25] M. Alhabeb , K. Maleski , B. Anasori , P. Lelyukh , L. Clark , S. Sin , Y. Gogotsi , Chem. Mater. 2017, 29, 7633.

[26] G. Zhuang , H. Zhang , H. Wu , Z. Zhang , L. Liao , Appl. Clay Sci. 2017, 135, 244.

[27] N. Mendel , D. Sîretanu , I. Sîretanu , D. W. F. Brilman , F. Mugele , J. Phys. Chem. C 2021, 125, 27159.

[28] J.‐M. Vallerot , X. Bourrat , A. Mouchon , G. Chollon , Carbon 2006, 44, 1833.

[29] A. Eckmann , A. Felten , A. Mishchenko , L. Britnell , R. Krupke , K. S. Novoselov , C. Casiraghi , Nano Lett. 2012, 12, 3925. 22764888 10.1021/nl300901a

[30] R. Trusovas , G. Račiukaitis , G. Niaura , J. Barkauskas , G. Valušis , R. Pauliukaite , Adv. Optical Mater. 2016, 4, 37.

[31] W. Xia , A. Mahmood , Z. Liang , R. Zou , S. Guo , Angew. Chem., Int. Ed. 2016, 55, 2650. 10.1002/anie.201504830 26663778

[32] R. Ryoo , S. H. Joo , S. Jun , J. Phys. Chem. B 1999, 103, 7743.

[33] J. Lee , S. Yoon , T. Hyeon , S. M. Oh , K. B. Kim , Chem. Commun. 1999, 2177.

[34] S. H. Joo , S. J. Choi , I. Oh , J. Kwak , Z. Liu , O. Terasaki , R. Ryoo , Nature 2001, 412, 169. 11449269 10.1038/35084046

[35] C. Liang , Z. Li , S. Dai , Angew. Chem., Int. Ed. 2008, 47, 3696. 10.1002/anie.200702046 18350530

