# Supplementary material for: End‐to‐End Pierced Carbon Nanosheets with Meso‐Holes
Source: Adv Sci (Weinh). 2024 Nov 25;12(3):2409546. doi: 10.1002/advs.202409546 (PMC11744651; doi:10.1002/advs.202409546)
Supplement: Supplementary file 1 — Supporting Information [file ADVS-12-2409546-s001.docx]

**Supplementary information**

**End-to-End Pierced Carbon Nanosheets with Meso-holes**

Minjun Kim,^1^ Hiroki Nara,^2^ Yusuke Asakura,^3^ Takashi Hamada,^3^ Peng Yan,^4^ Jacob Earnshaw,^1^ Meng An,^4^* Miharu Eguchi,^1,2^* Yusuke Yamauchi^1,3^*

^1^Australian Institute for Bioengineering and Nanotechnology (AIBN), The University of Queensland, Queensland 4072, Australia.

^2^Faculty of Science and Engineering and Waseda Research Institute for Science and Engineering.

^3^Department of Materials Process Engineering, Graduate School of Engineering, Nagoya University, Nagoya 464-8603, Japan.

^4^College of Mechanical and Electrical Engineering, Shaanxi University of Science and Technology, Xi'an 710021 China.

E-mail: anmeng@sust.edu.cn; eguchi@waseda.jp; y.yamauchi@uq.edu.au

**Methods**

Chemicals

Pluronic F127, dopamine hydrochloride, LiF and 1,3,5-trimethylbenzene were purchased from Sigma-Aldrich. Tris buffer was purchased from BioRad. Grapene oxide was purchased from XFNANO. Methyltriphenylphosphonium bromide, 3,4-dimethoxybenzaldehyde, and thioacetic acid were obtained from Tokyo Chemical Industry Co., Ltd. (Tokyo, Japan) and used without further purification. A 1.6 M solution of *n*-butyllithium (*n*-BuLi) in *n*-hexane was acquired from Kanto Chemical Co., Ltd and used as received. A 1 M solution of boron tribromide in dichloromethane, 2,2’-azobis(isobutyronitrile) (AIBN), dichloromethane (super-dehydrated), ethyl acetate, hexane, tetrahydrofuran (THF) (super-dehydrated), toluene (super-dehydrated), sodium hydroxide (NaOH), hydrochloric acid (HCl) were purchased from FUJIFILM Wako Pure Chemical Co., Ltd (Osaka, Japan). AIBN was used as received.

Synthesis of POMC

**MMT@mPDA**

To 100 mL mixture of deionized water (50 mL) and ethanol (50 mL), 1.0 g of F127 was added and fully dissolved by the continued stirring at 300 rpm. A volume of 5 mL of 1,3,5-trimethylbenzene (TMB) was then added and the solution was stirred for 2 hours at 300 rpm to form F127/TMB micelles. Next, 1.0 g of dopamine (DA) was added and dissolved at 300 rpm, and 0.1 g of montmorillonite (MMT) well-dispersed in 3 mL deionized water was added and stirred for 30 minutes at 300 rpm. Tris buffer [0.662 g dissolved in 4 mL mixture of deionized water (2.0 mL) and ethanol (2.0 mL)] was subsequently added to the mixture to initiate the polymerization. The mixture was left at 300 rpm for 12 hours for polymerization to obtain the sandwich-like 2D heterostructure consisting of MMT core and mesostructured polydopamine (mPDA) shell denoted as MMT@mPDA. The resulting solution was centrifuged to obtain the pellet of MMT@mPDA, which was washed with deionized water and ethanol several times with centrifugation. Upon the completion of washing, MMT@mPDA was re-dispersed in water and freeze-dried.

**MMT@mPDA-500**

MMT@mPDA was subjected to thermal curing at 500 °C for 2 hours under nitrogen flow. The ramping rate was fixed at 5 °C min^-1^. The thermally cured sample is denoted as MMT@mPDA-500.

**mPDA-500**

MMT@mPDA-500 was then subjected to *in situ* generated HF etching condition. Specifically, 100 mg of MMT@mPDA was dispersed in 20 mL of *in situ* generated HF (mixture of 20 mL 6 M HCl and 1.0 g LiF) for 12 hours to etch MMT and obtain exfoliated mPDA-500 layers.

**POMC**

mPDA-500 was subjected to thermal annealing at 900 °C for 2 hours under nitrogen flow. The ramping rate was fixed at 5 °C min^-1^. The thermally annealed sample is denoted as POMC.

Synthesis of PBMC

**GO@mPDA**

To 100 mL mixture of deionized water (50 mL) and ethanol (50 mL), 1.0 g of F127 was added and fully dissolved by the continued stirring at 300 rpm. A volume of 5 mL of TMB was then added and the solution was stirred for 2 hours at 300 rpm to form F127/TMB micelles. Next, 1.0 g of DA was added and dissolved at 300 rpm, and 0.1 g of graphene oxide (GO) well-dispersed in 3 mL deionized water was added and stirred for 30 minutes at 300 rpm. Tris buffer [0.662 g dissolved in 4 mL mixture of deionized water (2.0 mL) and ethanol (2.0 mL)] was subsequently added to the mixture to initiate the polymerization. The mixture was left at 300 rpm for 12 hours for polymerization to obtain the sandwich-like 2D heterostructure consisting of GO core and mPDA shell denoted as GO@mPDA. The resulting solution was centrifuged to obtain the pellet of GO@mPDA, which was washed with deionized water and ethanol several times with centrifugation. Upon the completion of washing, GO@mPDA was re-dispersed in water and freeze-dried.

**PBMC**

GO@mPDA was subjected to thermal annealing at 900 °C for 2 hours under nitrogen flow. The ramping rate was fixed at 5 °C min^-1^. The thermally annealed sample is denoted as PBMC.

**PBMC-HF**

GO@mPDA was subjected was subjected to thermal curing at 500 °C for 2 hours under nitrogen flow. The ramping rate was fixed at 5 °C min^-1^. The thermally cured sample was then subjected to *in situ* generated HF etching condition to obtain mPDA-500. HF-treated sample was washed until neutral pH was achieved, freeze-dried, and thermally annealed at 900 °C for 2 hours under nitrogen flow.

Synthesis of S-DA

Sulfur-containing monomer (S-DA) was synthesized in a four-step reaction according to our previous paper.^1^ 3,4-Dimethoxystyrene was synthesized via Wittig reaction in 64% yield. Dimethoxystyrene was synthesized several times and then combined together for the next reaction. Thioacetyl group was introduced through thiol-ene reaction to afford [2-(3,4-dimethoxyphenyl)ethyl]thioacetate in 85% yield. The deprotection of methoxy group with boron tribromide gave 87% yield of [2-(3,4-dihydroxyphenyl)ethyl]thioacetate. Thiol-ene reaction and deprotection proceeded quantitatively. 1,2-Dihydroxy-4-(2-mercaptoethyl)benzene was synthesized in 90% yield by hydrolysis. Sulfur-containing monomer was obtained as a white solid, total yield 43%. The detailed synthetic procedure is as follows:

**3,4-dimethoxystyrene**

In a dry 100 mL Schlenk flask, methyltriphenylphosphonium bromide (9.99 g, 28.0 mmol) was suspended in 50 mL of THF. A solution of 15.1 mL of *n*-BuLi (1.60 M in hexane) was slowly added to this suspension at 0 °C, and the mixture was stirred at room temperature for 1 h under a nitrogen atmosphere. Subsequently, 3,4-dimethoxybenzaldehyde (3.32 g, 20 mmol) in 20 mL of THF was added dropwise via syringe, and the reaction mixture was stirred at room temperature overnight under a nitrogen atmosphere. THF was evaporated under reduced pressure, and the residue was dissolved in dichloromethane and washed with water three times. The organic layer was dried over sodium sulfate, and the dichloromethane solution was evaporated under reduced pressure. The residue was purified by column chromatography on silica gel eluting with ethyl acetate–hexane (1:4) to afford 3,4-dimethoxystyrene as a colorless liquid (2.93 g, 64% yield). This compound was synthesized several times and then combined together for the next reaction.

**[2-(3,4-dimethoxyphenyl)ethyl]thioacetate**

In a dry 200 mL Schlenk flask, 3,4-dimethoxystyrene (8.03 g, 48.9 mmol), thioacetic acid (4.17 mL, 58.6 mmol), and AIBN (193 mg, 1.18 mmol) were dissolved in 80 mL of toluene. The resulting solution was heated at 80 °C for 6 h under a nitrogen atmosphere. After cooling to room temperature, toluene was evaporated under reduced pressure, and the residue was purified by column chromatography on silica gel eluting with ethyl acetate–hexane (1:4) to afford [2-(3,4-dimethoxyphenyl)ethyl]thioacetate as a white solid (9.98 g, 85% yield).

**[2-(3,4-dihydroxyphenyl)ethyl]thioacetate**

In a dry 500 mL two-necked flask, [2-(3,4-dimethoxyphenyl)ethyl]thioacetate (9.97 g, 41.5 mmol) was dissolved in 130 mL of dichloromethane, and the solution was cooled to −78 °C (dry ice/acetone) under a nitrogen atmosphere. A solution of boron tribromide (92 mL, 92 mmol) was slowly added dropwise. Then, the resulting solution was refluxed for 6 h. At room temperature, 150 mL of water was slowly added to quench excess boron tribromide, and the organic layer was separated. The aqueous layer was extracted with dichloromethane two times. The combined organic layer was dried over sodium sulfate, and the dichloromethane solution was evaporated under reduced pressure. The residue was passed through the silica gel eluting with ethyl-acetate-hexane (1:2) to afford [2-(3,4-dihydroxyphenyl)ethyl]thioacetate as a white solid (7.66 g, 87% yield). The monomer containing the solvent was used in the next reaction.

^1^H NMR (300 MHz, CDCl_3_): 6.76 (2H, m), 6.60 (1H, m), 5.93 (1.7H, brd), 3.04 (2H, t, *J* = 7.8 Hz), 2.71 (2H, t, *J* = 7.6 Hz), 2.32 (3H, s).

^13^C NMR (75 MHz, CDCl_3_): 30.7, 30.8, 35.1, 115.4, 115.6, 121.0, 132.8, 142.4, 143.7, 197.7.

IR (KBr): 3500–3200 (br), 3036, 2953, 2936, 1661, 1607, 1514, 1470, 1445, 1346, 1285, 1186, 1111, 961, 864, 814, 787 cm^–1^.

**1,2-dihydroxy-4-(2-mercaptoethyl)benzene (S-DA)**

In a 200 mL two-necked flask, [2-(3,4-dihydroxyphenyl)ethyl]thioacetate (7.45 g, 35.1 mmol) was dissolved in a mixture of 0.2 M NaOH solution (70 mL) and ethanol (70 mL). The resulting solution was stirred at room temperature for 24 h. Then, 14 mL of 1 M HCl solution was added for neutralization, and the resulting mixture was extracted with ethyl acetate three times. The combined organic layer was evaporated under reduced pressure. The residue was purified by column chromatography on silica gel eluting with ethyl acetate–hexane (1:2) to afford 1,2-dihydroxy-4-(2-mercaptoethyl)benzene as a white solid (5.40 g, 90% yield).

^1^H NMR (300 MHz, *d*_8_-THF): 7.81 (1H, s), 7.78 (1H, s), 6.59 (2H, m), 6.45 (1H, dd, *J* = 2.1, 8.1), 2.66 (4H, m), 1.58 (1H, t, *J* = 7.8).

^13^C NMR (75 MHz, *d*_8_-THF): 26.0, 40.0, 114.8, 115.3, 119.3, 131.4, 143.9, 145.3.

IR (KBr): 3500–3100 (br), 3020, 2968, 2938, 2567, 1613, 1530, 1443, 1370, 1275, 1211,1148, 1109, 951, 872, 814, 791 cm^–1^.

Synthesis of S-POMC

To 100 mL mixture of deionized water (50 mL) and ethanol (50 mL), 1.0 g of F127 was added and fully dissolved by the continued stirring at 300 rpm. A volume of 5 mL of 1,3,5-trimethylbenzene (TMB, Sigma-Aldrich) was then added and the solution was stirred for 2 hours at 300 rpm to form F127/TMB micelles. Next, 1.0 g of DA and 0.3 g of S-DA were added and dissolved at 300 rpm, and 0.1 g of montmorillonite (MMT) well-dispersed in 3 mL deionized water was added and stirred for 30 minutes at 300 rpm. Tris buffer [0.861 g dissolved in 4 mL mixture of deionized water (2.0 mL) and ethanol (2.0 mL)] was subsequently added to the mixture to initiate the polymerization. The mixture was left at 300 rpm for 36 hours for polymerization to obtain the sandwich-like 2D heterostructure denoted as MMT@mPDA-S. The resulting solution was centrifuged to obtain the pellet of MMT@mPDA-S, which was washed with deionized water and ethanol several times with centrifugation. Upon the completion of washing, MMT@mPDA-S was re-dispersed in water and freeze-dried. Nitrogen and sulfur co-doped POMC (N/S-POMC) was obtained by following the same procedures for POMC from MMT@mPDA.

Characterizations

**Microscopy analyses**

For analysis, the samples were suspended in ethanol by ultrasound for 60–240 s, depending on their dispersing ability. FE-SEM was performed with a JEOL JSM-7100F at 2 kV. TEM was carried out at 120 kV with a Hitachi HT7700. TEM, STEM and EDS mapping were performed at 200 kV with Hitachi HF5000. Bright-field transmission electron microscopy (BF-TEM) and scanning TEM images were obtained using a chromatic aberration (Cs) corrected TEM (Grand ARM300F, JEOL) at an acceleration voltage of 160 kV.

**Height profile analyses**

AFM was conducted with a Bruker Multimode 8-HR in the tapping (contact) mode at 1 Hz. For the AFM measurements, a droplet of the sample dispersion in ethanol was placed on a silicon wafer (1 x 1cm) and dried, which was used for the measurement. The height profile of the electrode was obtained by KLA P-7 Stylus Profiler.

**Surface area and porosity analyses**

N_2_ adsorption-desorption isotherms were measured by BELSORP Mini X surface area and pore size analyzer at liquid nitrogen temperature (77 K). Prior to the measurement, the sample was degassed under vacuum at 120 °C for 6 hours.

**Spectroscopy analyses**

XRD was performed with Bruker D8 Advance powder XRD under Cu-Kα radiation at 40 kV and 40 mA. XPS was performed with Kratos Axis Supra Plus XPS using mono-Al Kα (1486.6 eV) X-rays. For XPS measurement, the sample was mounted on the conductive tape to minimize the charging. Adventitious carbon peak at 284.8 eV was used as a reference binding energy. Raman spectroscopy was performed with Renishaw Raman microscope and spectrometer at an excitation wavelength of 532 nm.

**Finite element simulation analysis**

Finite element simulations were obtained by COMSOL software. The simulations involved the coupled utilization of the transport diluted species module and the electrostatics module to model the ion transport behavior.

**ζ-potential analysis**

ζ-potential analysis was conducted with Otsuka Electronics ELSZ-2000 with a flow cell.

**Monomer analysis**

^1^H and ^13^C nuclear magnetic resonance (NMR) measurements were conducted on a Bruker 300 MHz NMR spectrometer in deuterated chloroform (CDCl_3_) or deuterated tetrahydrofuran (*d*_8_-THF), using residual chloroform or THF as a reference. Fourier-transform infrared spectroscopy (FT-IR) was performed with KBr methods on Shimazu IRAffinity-1S.

**Electrochemical analyses**

All electrochemical measurements were carried out using CHI760 (CH Instruments) under three electrode systems. The counter electrode and reference electrode used were a carbon rod and Hg/HgO electrode saturated with 1 M NaOH, respectively. The rotating ring disk electrode (Pt ring with glass carbon disk RRDE, ALS) was used as the working electrode, and the area is 0.126 cm^2^. The catalyst ink was prepared by mixture of 5 mg of catalyst and 50 µl of Nafion ionomer solution (5 wt. % in mixture of lower aliphatic alcohols and 45% of water, Sigma-Aldrich) in 950 µl of 1:2 deionized water/2-isopropanol (Sigma-Aldrich) with 1 h sonication. The catalyst loading of carbon materials in alkaline and acid electrolyte measurements was 0.2 mg cm^-2^. Prior to the electrochemical measurement, the RRDE is pretreated by means of cyclic voltammetry (CV) with a scan rate of 100 mV s^-1^ between 0.01 and 1.15 V vs. RHE. The electrochemical measurements were conducted in O_2_/N_2_-saturated 0.1 M KOH electrolytes. CV was performed at various scan rates from 10 to 200 mV s^-1^, and linear sweep voltammetry (LSV) was performed at a rotating speed of 1600 rpm with a scan rate of 10 mV s^-1^. The LSV measurements were also conducted at different rotating speeds from 400 to 2025 rpm. The non-Faradaic capacitive effect was compensated for by subtracting the current measured in N_2_ saturated condition. Electrochemical impedance spectroscopy (EIS) was performed at open circuit potential without rotating with an amplitude of 10 mV between 100 kHz and 10 m Hz. Also, *operando* EIS was conducted with an amplitude of 10 mV between 100 kHz and 0.01 Hz at various operating potentials with 1600 rpm rotating in O_2_ saturated condition.

All the potentials in the measurement were converted to potentials *vs*. the reversible hydrogen electrode (RHE) according to the following equation:

*E*_RHE_ = *E*_Hg/HgO_ + 0.0591 $\times$*pH* + 0.118

Kinetic current density was calculated according to the Koutecky-Levich equation:

$$\frac{1}{j}=\frac{1}{j_{L}}+\frac{1}{j_{K}}$$

where, *j* is the measured current density, *j*_k_ and *j*_L_ are the kinetic and diffusion-limiting current densities, respectively.

Double layer capacitance by EIS (*C*_dl_EIS_), that is a static condition was calculated from a Nyquist plot. The complex capacitance *C*(*ω*) is represented as following:^2^

$$C\left( \omega\right)=\frac{1}{j\omega Z\left( \omega\right)}$$

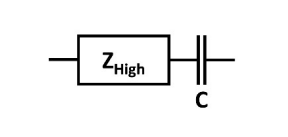
where *ω* is angular frequency (rad s^-1^) and *Z*(*ω*) is complex impedance as a function of angular frequency. Supplementary note: To calculate *C*_dl_EIS_, the raw data from low frequency region of EIS are used. The equation “*C*(*ω*)=1/*jωZ*(*ω*)” can be represented to the following equivalent circuit:

The impedance in the high-frequency range, which includes electric and ionic resistances and capacitance in series, is represented by the equivalent circuit shown above. In the low frequency domain, however, *Z*_High_'s impedance is constant, allowing *C*_dl_EIS_ to be easily estimated from the raw data.

The ion diffusion coefficient is obtained according to the following: ^3^

$D_{{OH}^{-}}=\frac{R^{2}T^{2}}{2A^{2}n^{4}F^{4}C^{2}\sigma^{2}}$ (6)

where $R$ is the gas constant (8.314 J K^−1^ mol^−1^ ), $T$ is the room temperature (298 K ), $A$ is the the surface area of the electrode used for testing (0.19625 cm^2^ ), $n$ is the number of transferred electrons (4), $F$ is the Faraday constant (96485 C mol^−1^), $C$ is the concentration of OH^−^ (0.1 mmol cm^−3^). According to $Z' = R_{s} + R_{ct} + \sigma\omega^{-1/2}$, it can be concluded that $\sigma$ is the slope of the plot of $Z'$.

Charge transfer resistances and mass transport resistances were calculated from *operando* EIS data obtained at various potentials using a transmission line model (TLM) represented below.


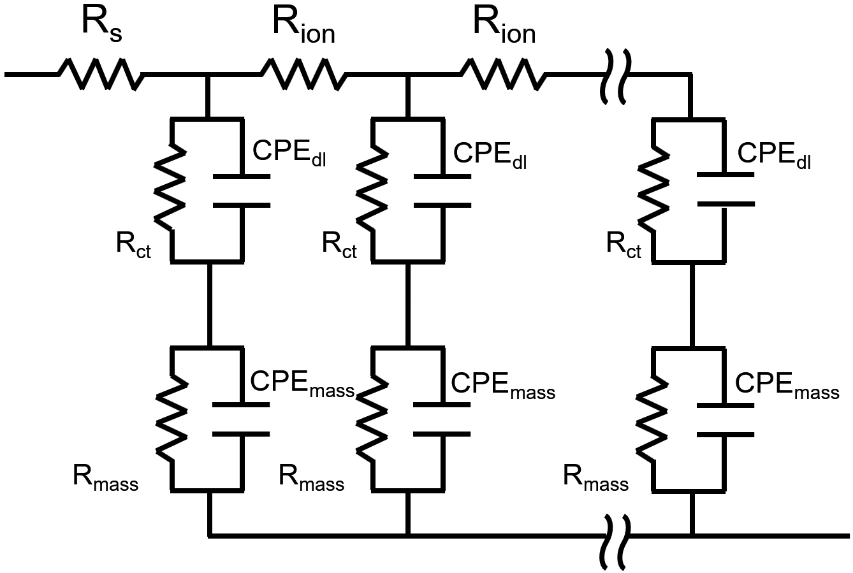


Although, this model was formally applied to the fitting analysis to minimize a fitting error on charge transfer resistance because Nyquist plots in high frequency region was noisy, the difference of fitting results between with and without using TLM is little on the charge transfer resistance and the mass transport resistance: ^4^ The equivalent circuit without using TLM is a series circuit composed of *R*_s_ and the parallel circuits of *R*_ct_/CPE_dl_ and *R*_mass_/CPE_mass_.


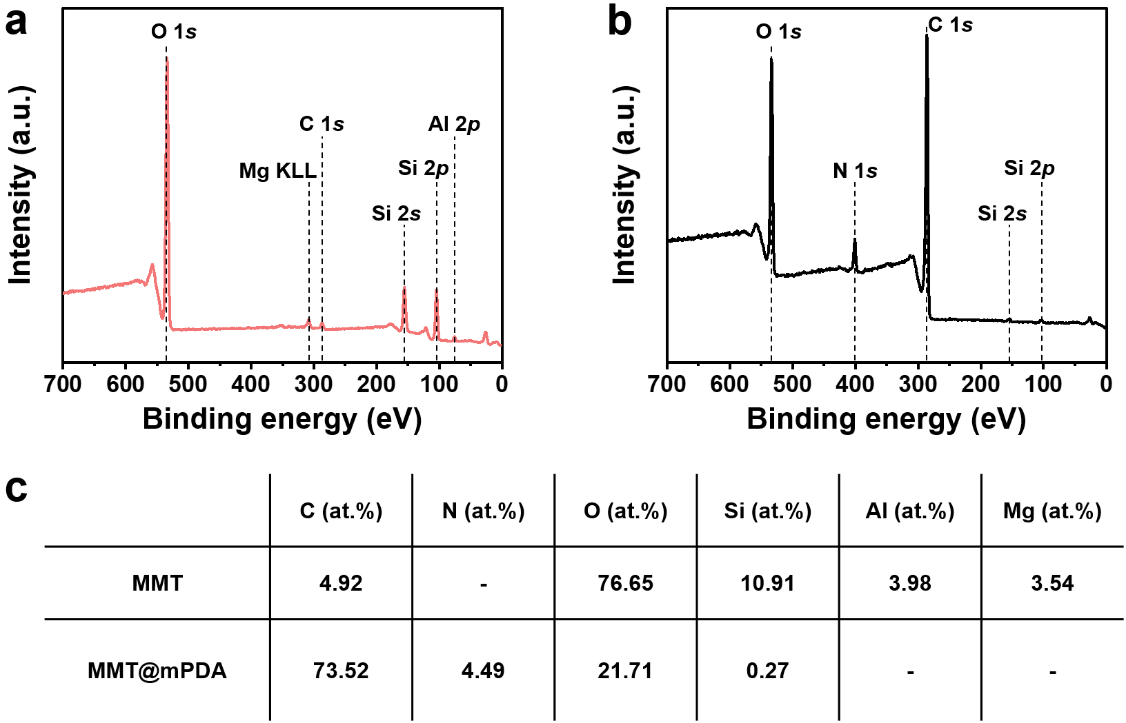


**Supplementary Fig. S1.** Survey XPS spectra of (a) MMT and (b) MMT@mPDA. (c) Table showing the elemental contents of MMT and MMT@mPDA.


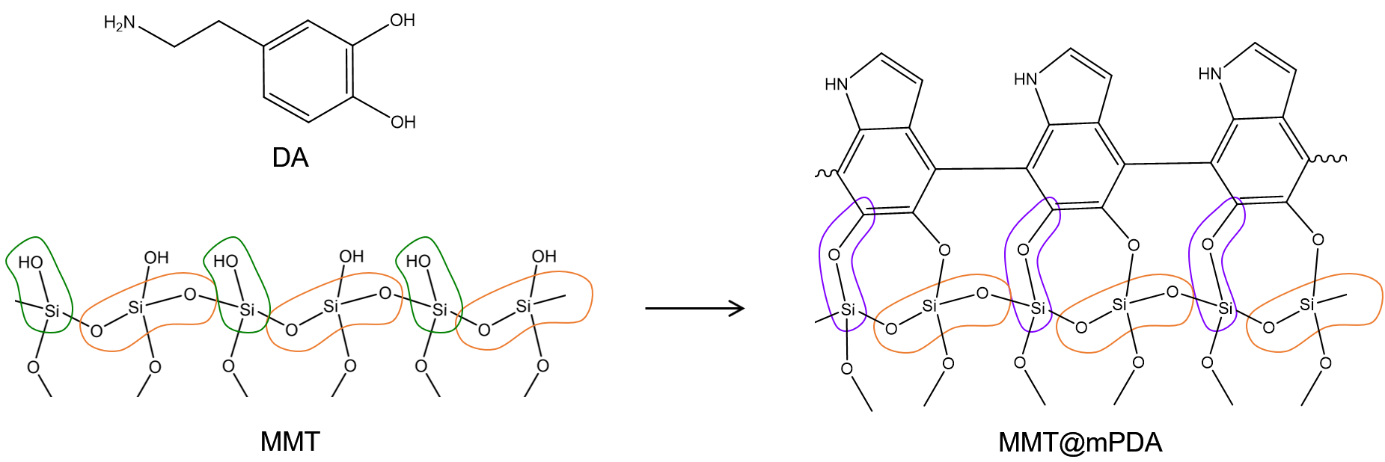


**Supplementary Fig. S2.** Molecular structure of DA, MMT and MMT@mPDA corresponding to XPS analysis.


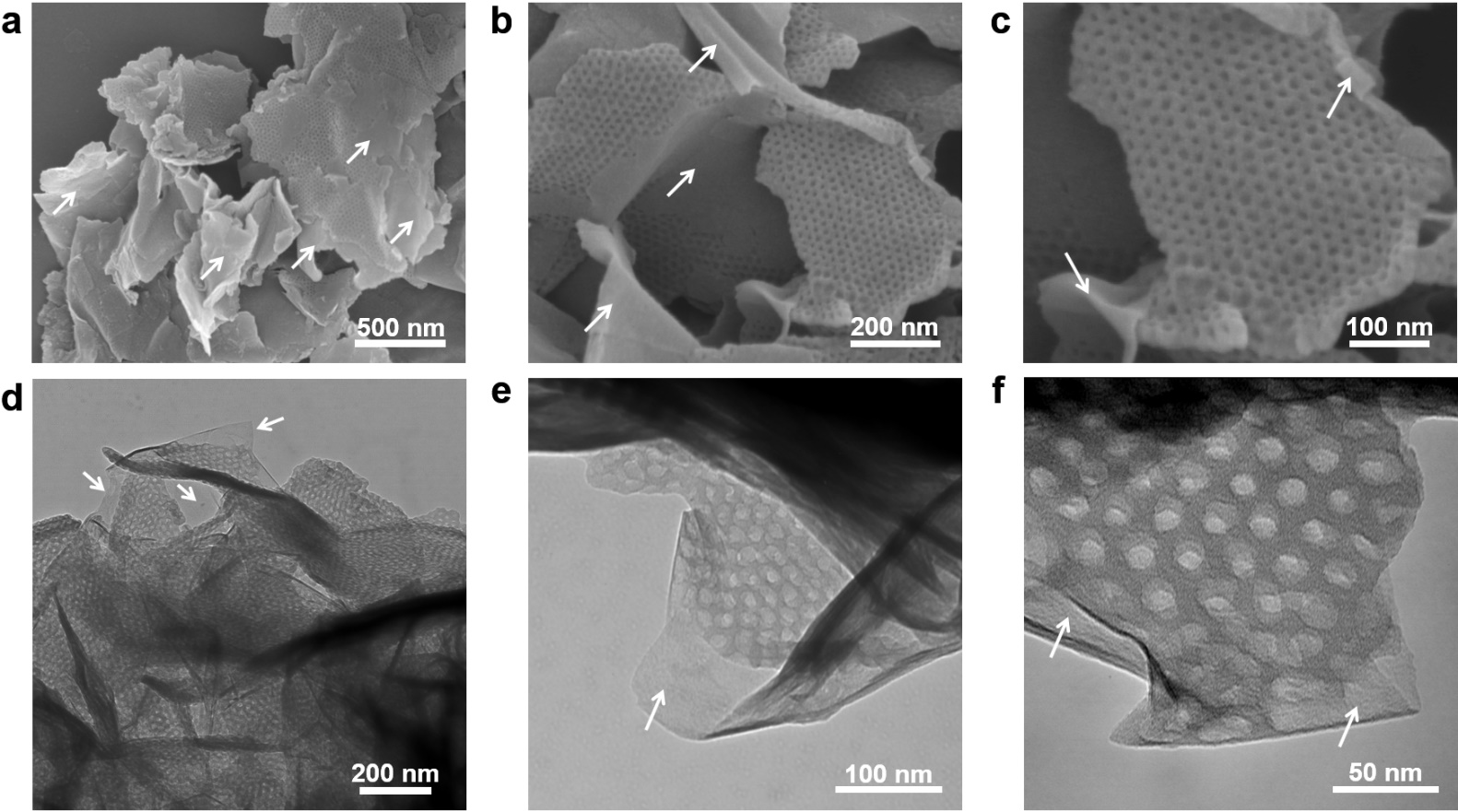


**Supplementary Fig. S3.** (a-c) SEM and (d-f) TEM images of MMT@mPDA-500. White arrows indicate the re-exposed part of MMT after the thermal curing.


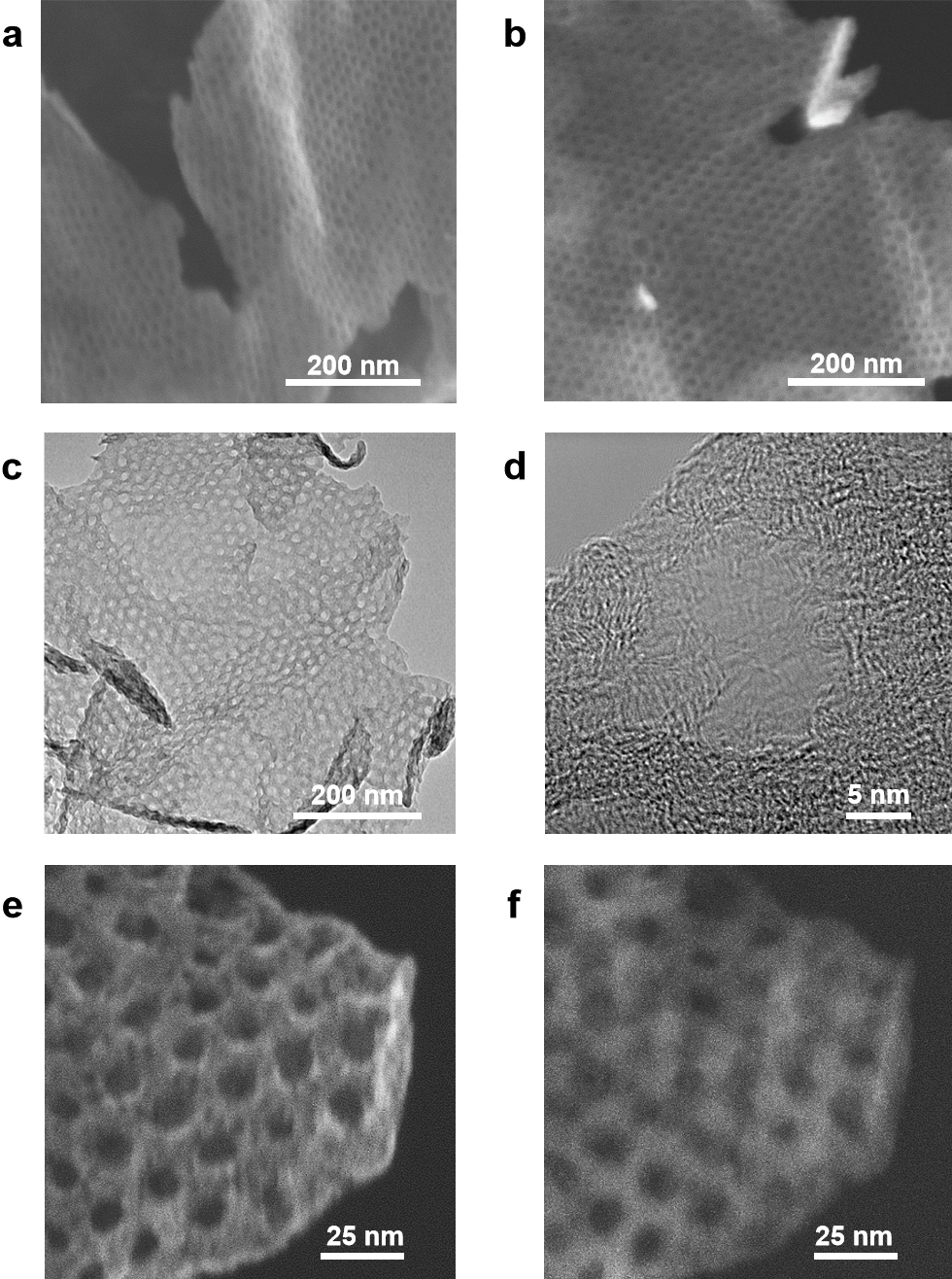


**Supplementary Fig. S4.** (a, b) SEM and (c, d) TEM images of POMC. (e) SE-STEM and (f) DF-STEM images of POMC.


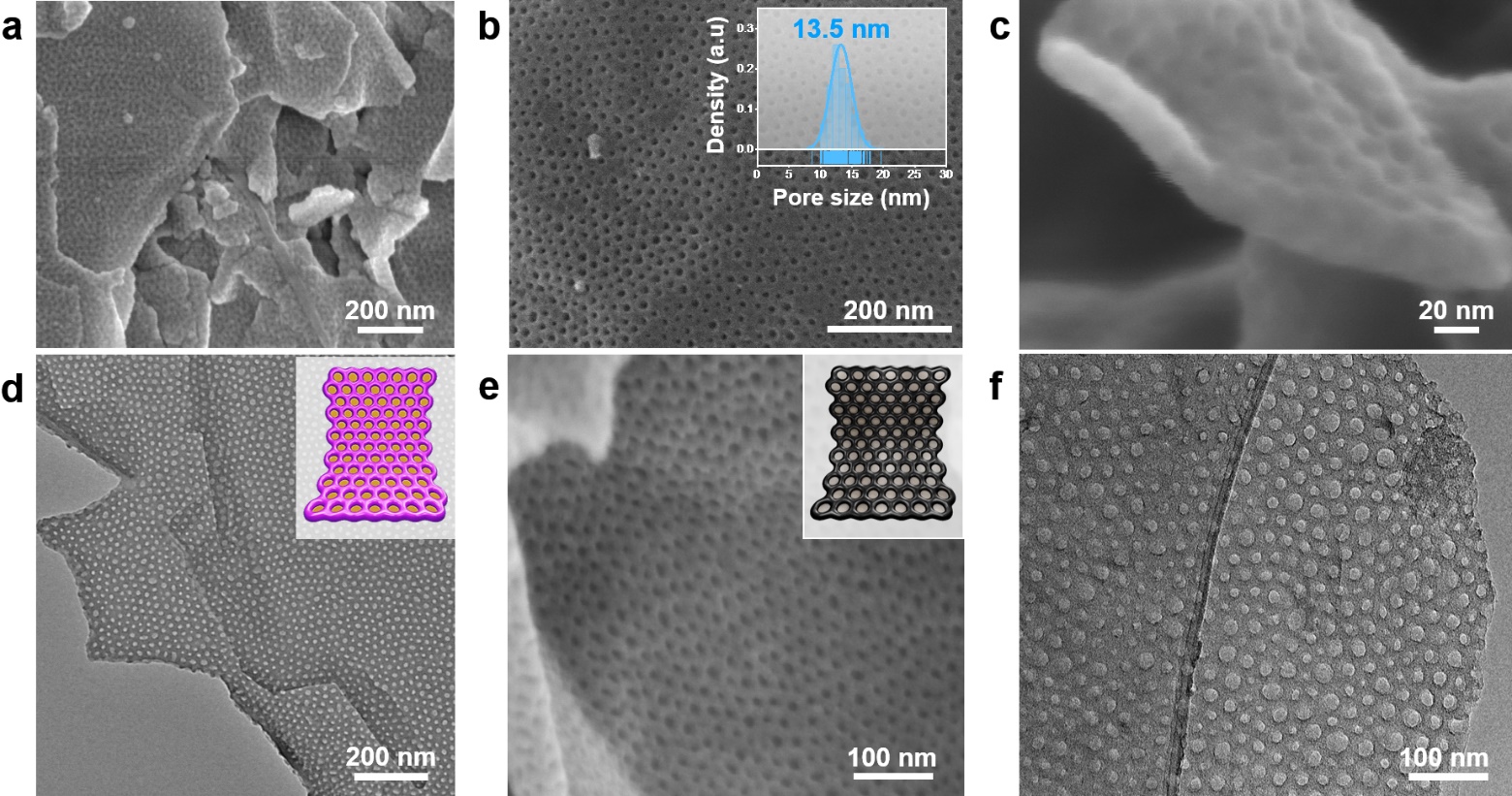


**Supplementary Fig. S5.** (a-c) SEM and (d) TEM images of GO@mPDA. (e) SEM and (f) TEM images of PBMC.

Supplementary note to Supplementary Fig. S5.

The inset of (b) represents the pore size distribution of GO@mPDA obtained by ‘ImageJ’ software).


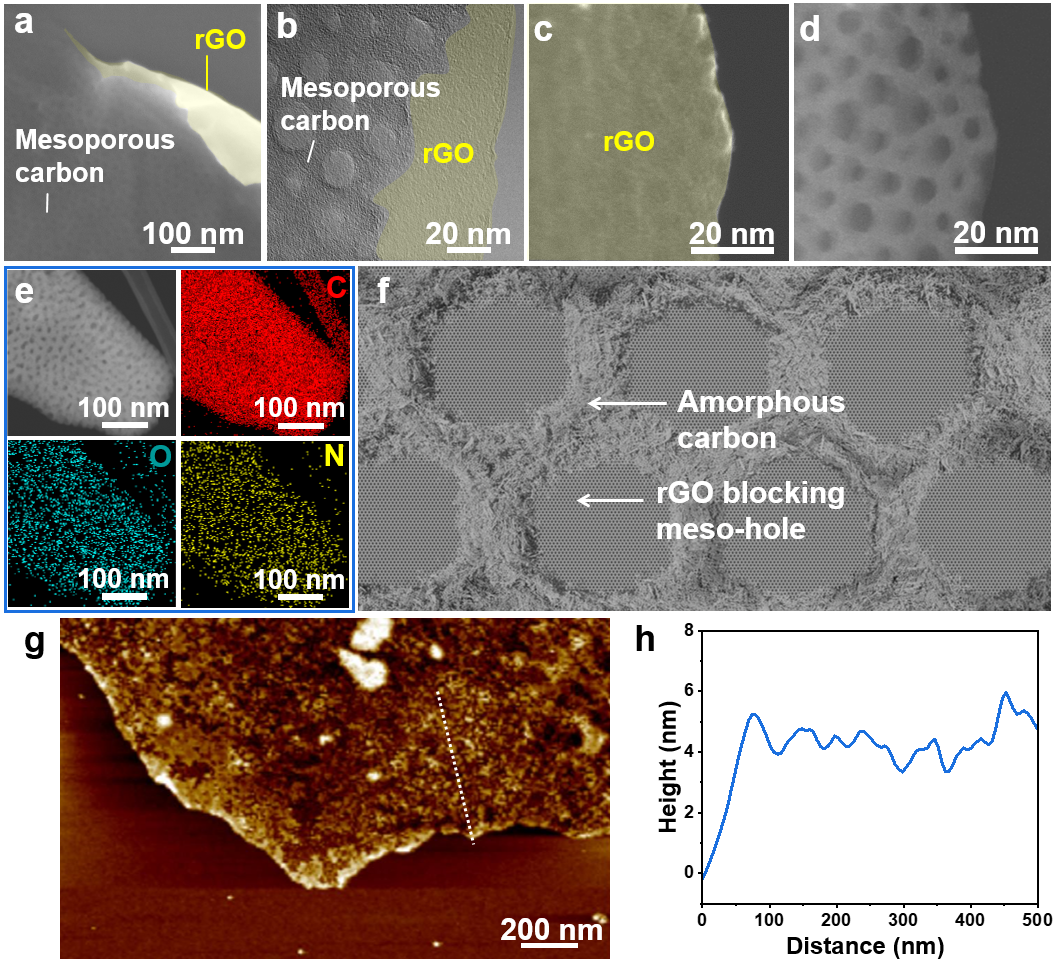


**Supplementary Fig. S6.** (a) SEM, (b) TEM, (c) SE-STEM, (d) DF-STEM and (e) EDS images of PBMC. (f) Schematic description of PBMC. (g) AFM image and (h) the corresponding height profile of PBMC. Yellow-highlighted area in (a-c) indicates the exposed rGO of PBMC.

Supplementary note to Supplementary Fig. S6.

PBMC with exposed rGO is specifically selected for the STEM observation to better visualize the blocked meso-holes. SE-STEM and DF-STEM images of PBMC demonstrate a marked difference in terms of the visualization of meso-holes due to the blocking of the meso-holes by rGO (Supplementary Figs. S6c, d). Typically, the SE-STEM image of PBMC shows that the meso-holes are beneath the sheet of rGO whereas the DF-STEM image clearly shows the meso-holes in the 2D structure because the rGO is much thinner than the meso-holey carbon layer beneath, hence is not able to produce sufficiently visible contrast. In addition, the elemental mapping for C atoms does not tend to follow the pore walls of PBMC (Supplementary Fig. S6e), therefore offering further evidence that the meso-holes are perpendicularly blocked as described in the scheme (Supplementary Fig. S6f).


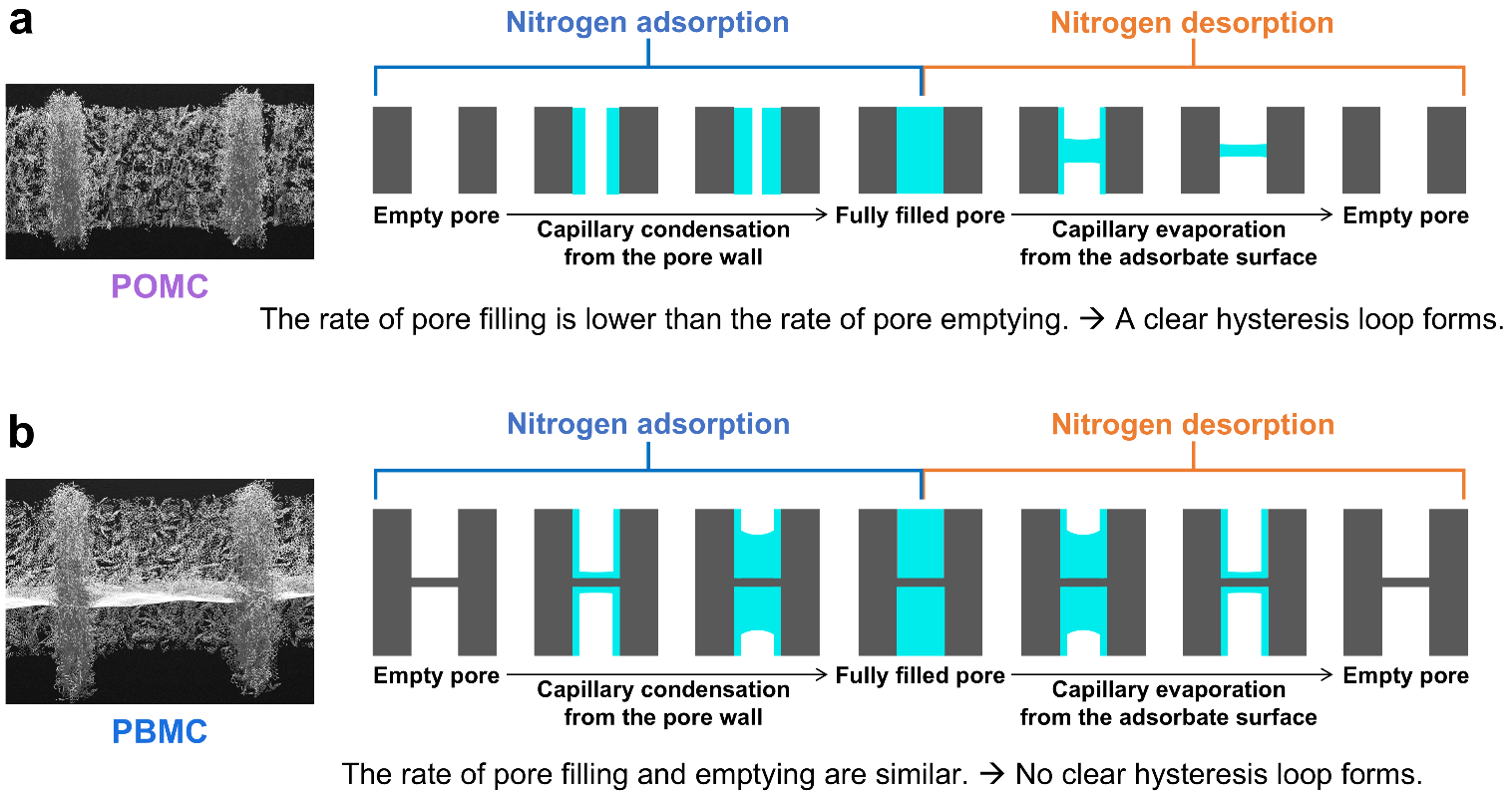


**Supplementary Fig. S7.** The proposed mechanism of nitrogen adsorption and desorption to/from (a) POMC and (b) PBMC.

Supplementary note to Supplementary Fig. S7.

For POMC, the adsorption process involves capillary condensation occurring from the pore wall and gradually inward to the center. On the contrary, the desorption process involving capillary evaporation occurs from the surface of condensed nitrogen in the pore, therefore, allowing the evaporation to happen more readily than condensation and the formation of hysteresis (Supplementary Fig. S7a). For PBMC, however, the rate of capillary condensation and evaporation during adsorption and desorption processes, respectively, are similar as the surface of rGO is also involved in nitrogen adsorption along with the pore wall. Consequently, the nitrogen adsorption-desorption isotherm of PBMC does not present hysteresis (Supplementary Fig. S7b).


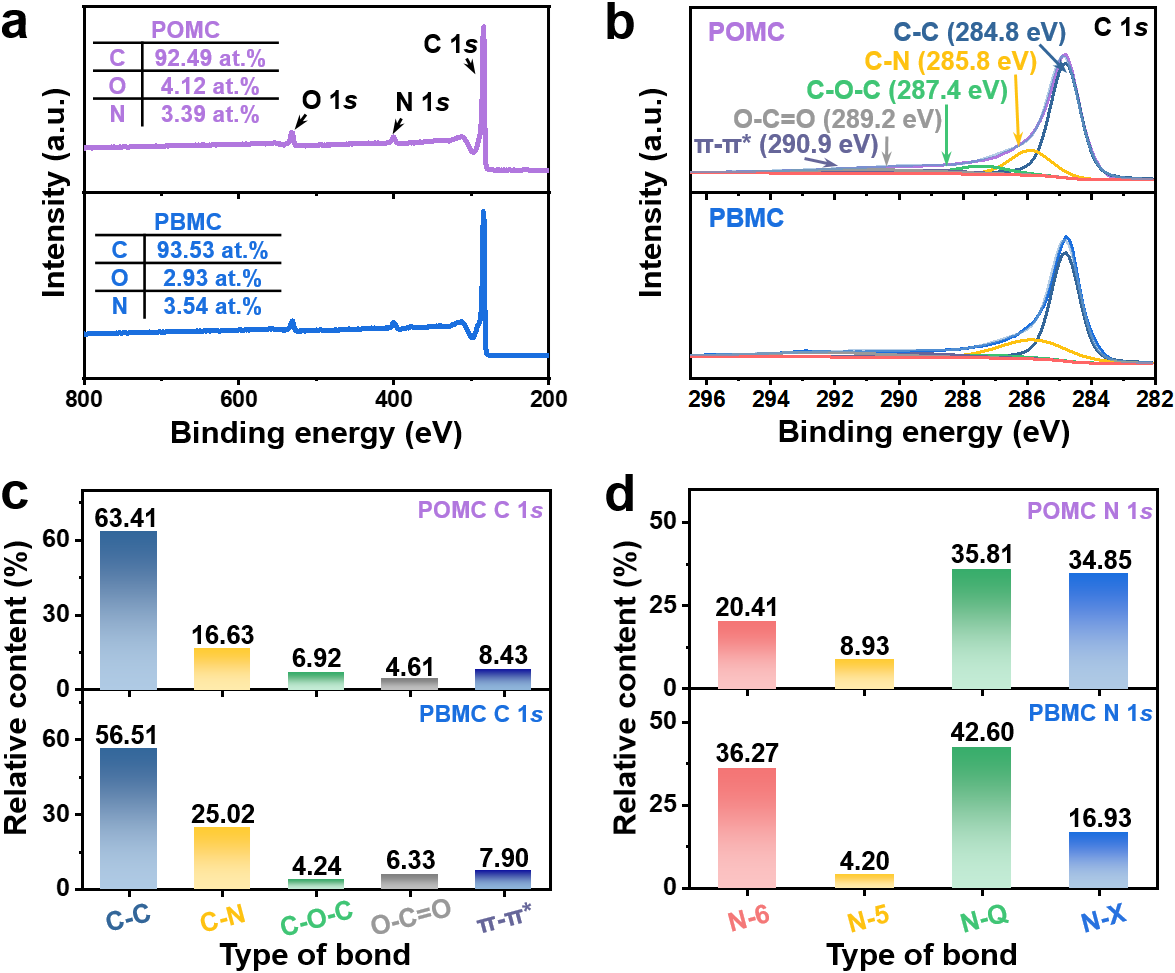


**Supplementary Fig. S8.** (a) Survey XPS spectra of POMC and PBMC and their chemical compositions. (b) High resolution XPS spectra of POMC and PBMC for C 1*s*. Relative content of bond types present in POMC and PBMC for (c) C 1*s* and (d) N 1*s*.


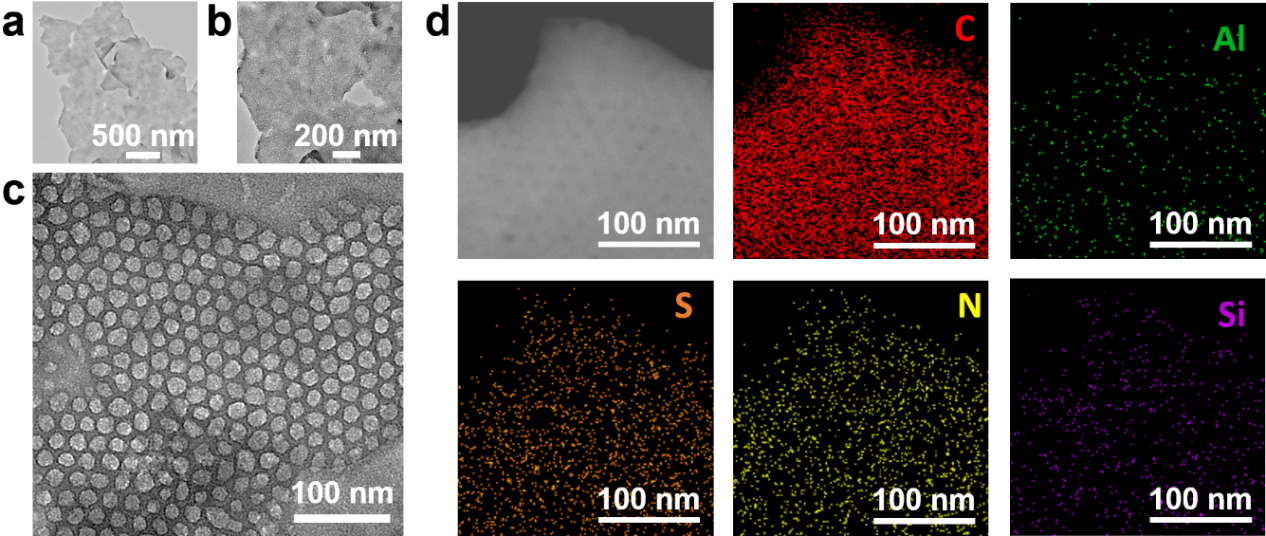


**Supplementary Fig. S9.** (a-c) TEM images of MMT@mPDA-S. (d) STEM-EDS images of MMT@mPDA-S.


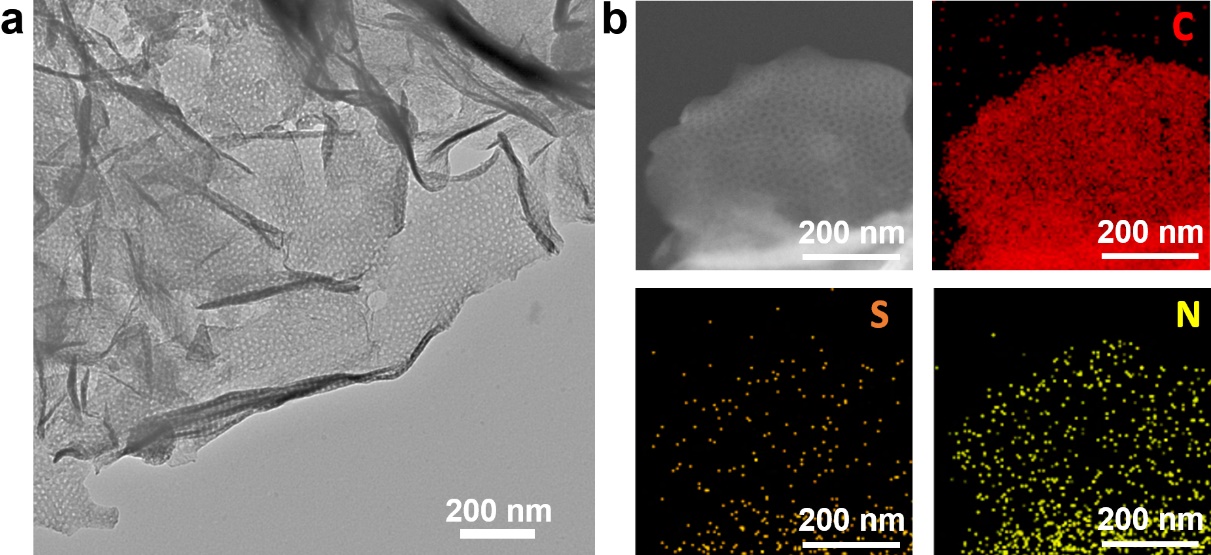


**Supplementary Fig. S10.** (a) TEM image of S-POMC. (d) STEM-EDS images of S-POMC.


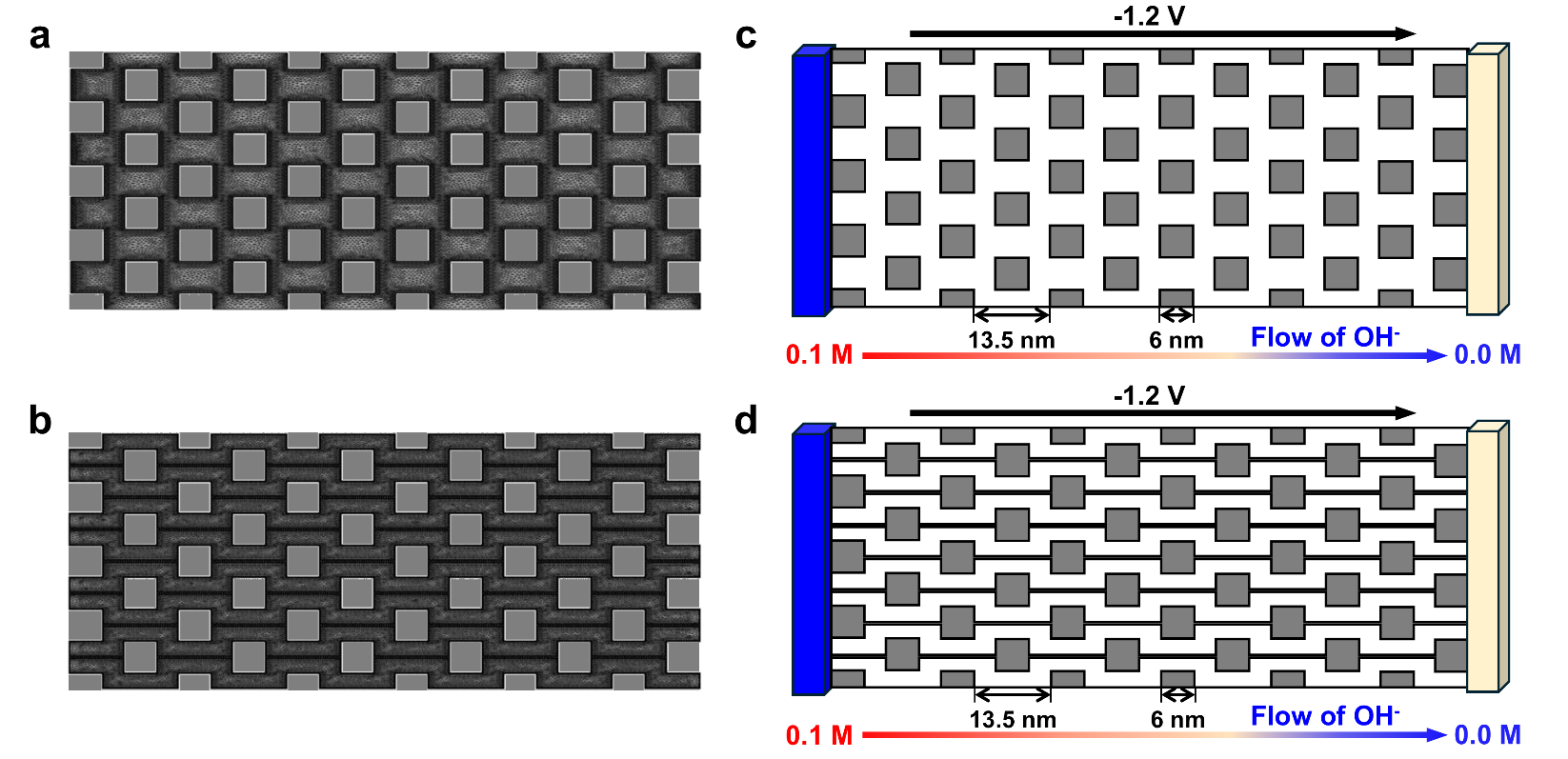


**Supplementary Fig. S11.** schematic description of the mesh quality and numerical setup used in finite element calculation of (a) POMC and (b) PBMC stacked in 7 layers for electrodes. The mesh numbers of POMC and PBMC are 97928 and 114960, respectively. Schematic description of (c) POMC and (d) PBMC electrode systems with 0.1 M and 0.0 M OH^-^ solution are applied at the left and right sides of systems, respectively. The voltage of -1.2 V was employed along the direction of concentration gradient. The diameter and space distance of mesopore are 13.5 nm and 6 nm, respectively.

Supplementary note to Supplementary Fig. S11.

The concentration distribution of OH^-^ across PBMC and POMC under molar concentration field and electric fields. To investigate the electrolyte diffusion behavior across different porous structures, the concentration and streamline distribution of electrolyte were calculated using finite element simulations implemented by COMSOL software. The interlayer structures of POMC and PBMC are simplified as the 2D models, shown in Supplementary Fig. S11. The periodic boundary conditions are applied in two in-plane directions. The simulations involved the coupled utilization of the transport diluted species module and the electrostatics module to model the ion transport behavior within POMC and PBMC.

The OH^-^ solution transport in electrodes consisting of 7 stacks of POMC and PBMC in zigzag can be described by Nernst-Planck equation:

$\frac{\partial c_{i}}{\partial t}+\nabla\cdot(-D_{i}\nabla c_{i}-\frac{Fz_{i}D_{i}c_{i}}{RT}\nabla\boldsymbol{\phi})=0$ (S1)

where *c_i_* is the molar concentration of OH^-^ solution, *D_i_* is the diffusion coefficient, *z*_i_ denotes the ion charge, *F* is the Faraday constant, *R* represents the gas constant, *T* stands for absolute temperature, and $\boldsymbol{\phi}$ represents the electric potential which is described by the Poisson equation. The OH^-^ solution diffusion inside two types of electrodes was driven by the concentration gradient and electrical field, where 0.1 M OH^-^ solution was applied at the left side of simulation model and a voltage of 1.2 V was applied along the concentration gradient direction. The right side of simulation model is set as the outflow boundary condition. The upper and lower boundaries of two types of models are set as continuous periodic boundary conditions.

The Poisson equation was used to describe the external electric field and surface charge distribution and can be written as,

$\nabla\cdot\left( \varepsilon\nabla\boldsymbol{\phi} \right)=-F\sum_{i} z_{i}c_{i}$ (S2)

where *c_i_* is the molar concentration of OH^-^ solution, *F* denotes the Faraday constant, *z*_i_ denotes the ion charge, $\varepsilon$ represents the dielectric constant and $\varepsilon= \varepsilon_{0}\cdot\varepsilon_{r}$ with the vacuum permittivity of *ε_0_ =* 8.854*×*10^-12^ F m^-1^ and the 0.1 M OH^-^ solution relative dielectric constant of ε_r_ =3.3. $\phi$ denotes the electrical potentials, where the boundary condition on the left is written as,

$\nabla_{\perp}\boldsymbol{\phi}=0$ (S3)

Meanwhile, the boundary condition of external electric field on the right $\phi_{1}$= -1.2 V. The electrical field $\phi_{2}$ is determined by the surface charge density of materials, given by Gaussian law:

$\nabla\cdot\left( \varepsilon\nabla\boldsymbol{\phi}_{\boldsymbol{2}} \right)=\rho$ (S4)

where $\rho$ = 1.6022×10^-19^ C m^-2^ is the surface charge density of materials. The initial concentration of KOH at the left end is set as 0.1 M while the one at right end is for 0 M. The normal flux inside POMC and PBMC models can be expressed as,

$\nabla_{\perp}J_{i}=0$ (S5)

$J_{i}=-D_{i}\nabla c_{i}-\frac{Fz_{i}D_{i}c_{i}}{RT}\nabla\boldsymbol{\phi}$ (S6)

The transient simulations were performed to obtain the concentration distribution of OH^-^ solution in POMC and PBMC in zigzag at different simulation time.


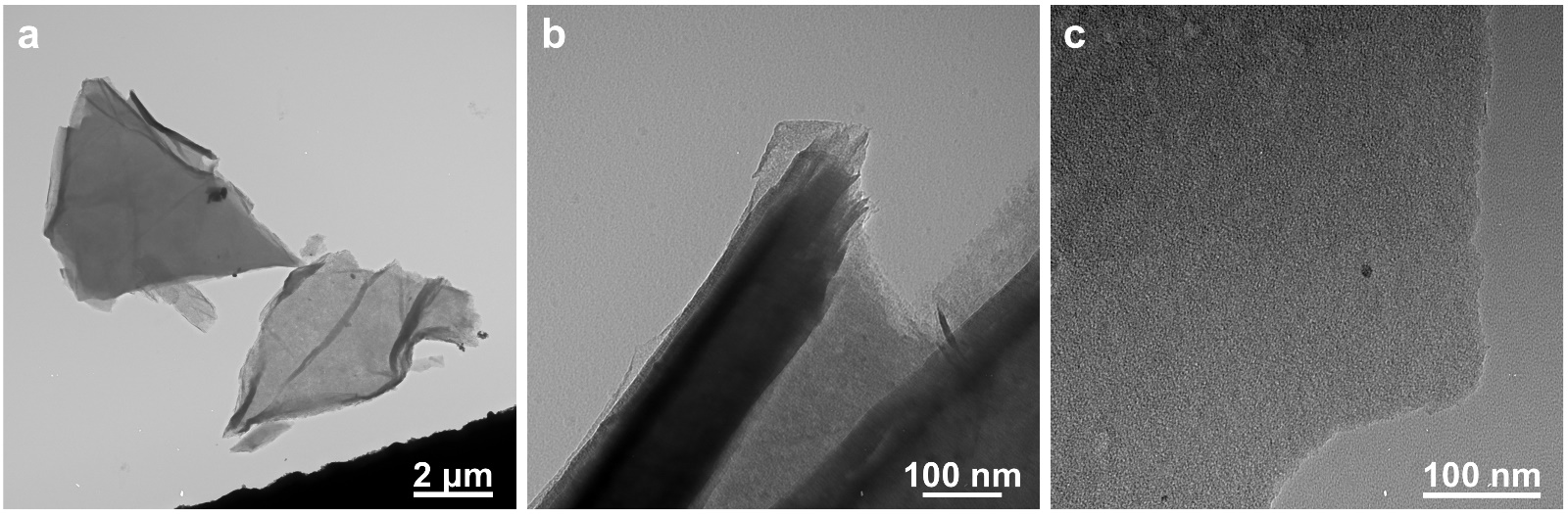


**Supplementary Fig. S12.** (a-c) TEM images of rGO.


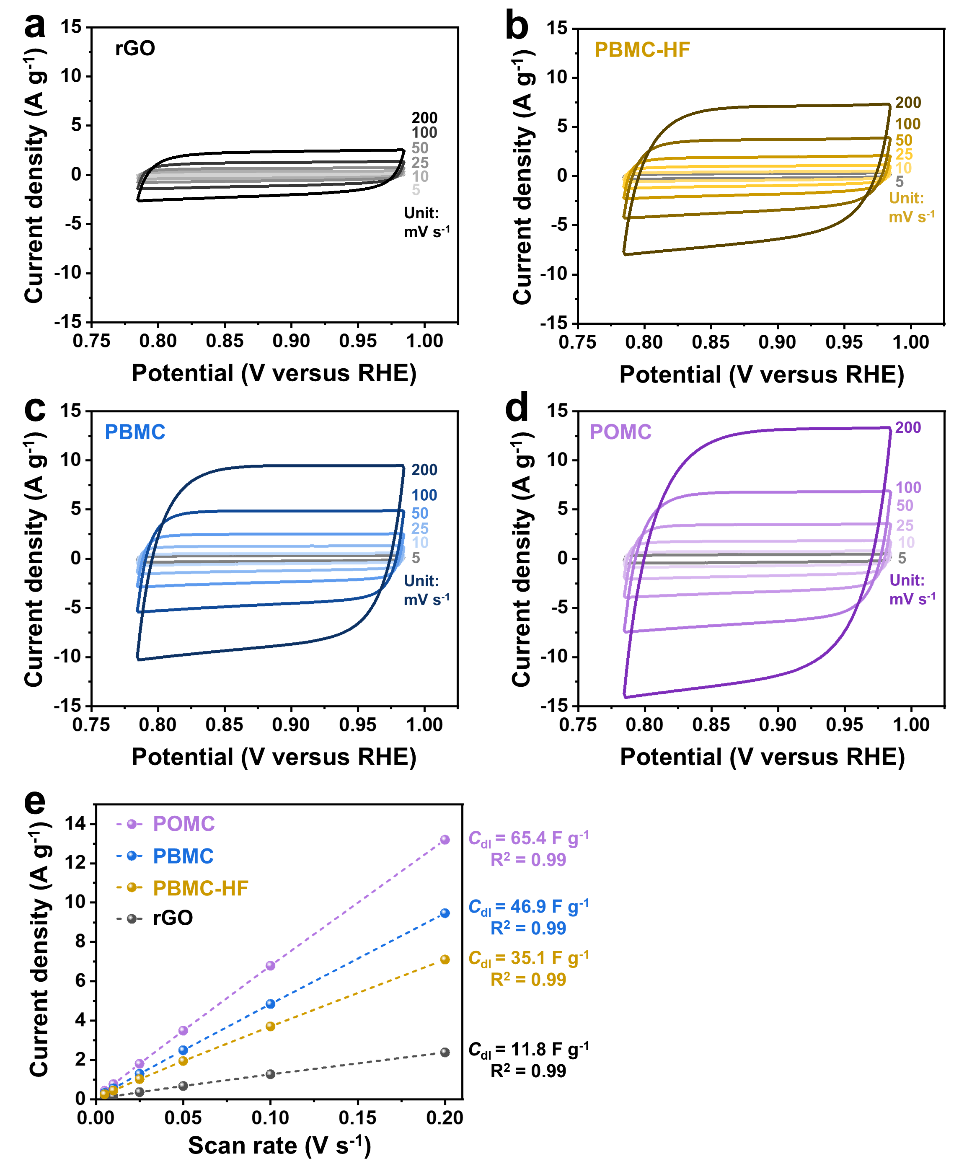


**Supplementary Fig. S13.** CV curves of (a) rGO, (b) PBMC-HF, (c) PBMC and (d) POMC at different scan rates from 5 to 200 mV s^-1^. (e) Linear fitted lines of anodic currents measured at specific scan rates from 5 to 200 mV s^-1^.

Supplementary note to Supplementary Fig. S13.

To calculate the double-layer capacitance (*C*_dl__CV) that is a dynamic condition of carbon sample, CV curves were first obtained at a non-faradaic region with scan rates at 5, 10, 25, 50, 100 and 200 mV s^-1^ (Supplementary Fig. S8a, b). The anodic currents at 0.90 V of the CV curves were plotted as a function of scan rates, and the slope of linear fitted line was obtained (Supplementary Fig. S8c). The slope indicates *C*_dl__CV of the carbon sample. Typically, *C*_dl__CV values of rGO, PBMC-HF, PBMC and POMC are 11.8 F g^-1^, 35.1 F g^-1^, 46.9 F g^-1^ and 65.4 F g^-1^, respectively (R-values of linear fitted lines for rGO, PBMC-HF, PBMC and POMC are 0.99).


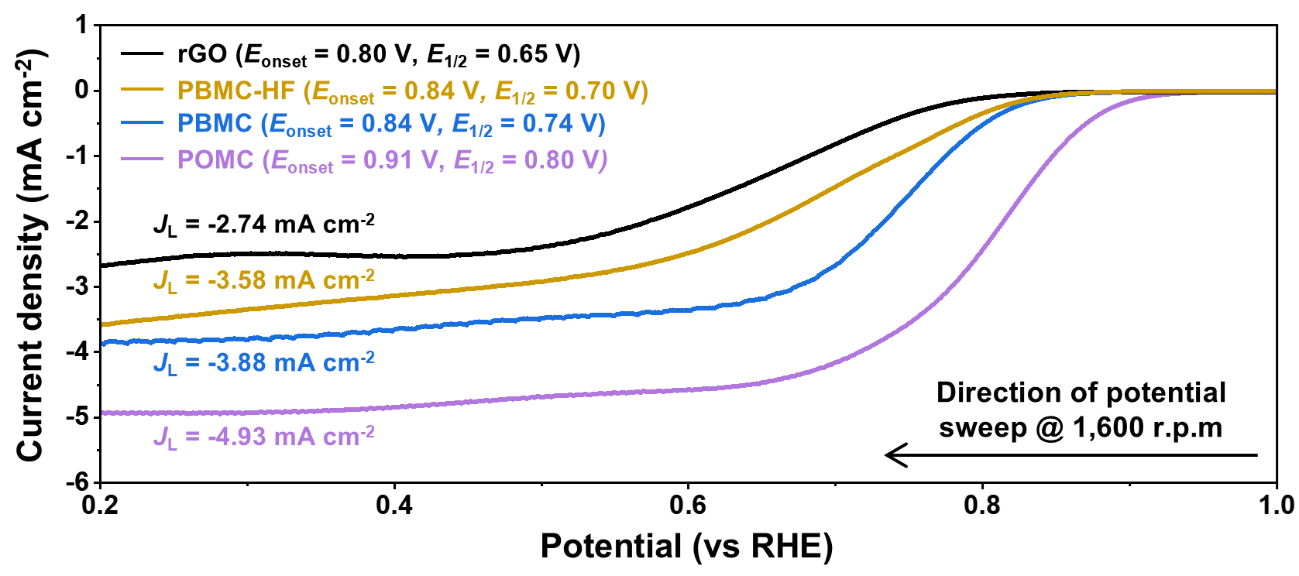


**Supplementary Fig. S14.** LSV curves of rGO, PBMC-HF, PBMC and POMC at 1600 rpm in O_2_ saturated 0.1 M KOH electrolyte at 10 mV s^-1^.

Supplementary note to Supplementary Fig. S14.

According to the LSV curves, POMC significantly outperforms rGO, PBMC-HF and PBMC in terms of onset potential (*E*_onset_), half-wave potential (*E*_1/2_) and maximum limiting current density (*j*_L_) for electrochemical reduction of oxygen as indicated in the plot.


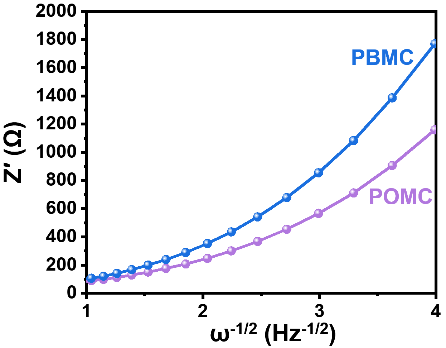


**Supplementary Fig. S15.** Plot of Z′ against ω^−1/2^ based on the EIS recorded at open circuit potential for PBMC and POMC.

Supplementary note to Supplementary Fig. S15.

Diffusion coefficient of OH^-^ is calculated from the following equation:

$D_{{OH}^{-}}=\frac{R^{2}T^{2}}{2A^{2}n^{4}F^{4}C^{2}\sigma^{2}}$

where $R$ is the gas constant, $T$ is the temperature, $A$ is the the surface area of the electrode used for testing, $n$ is the number of transferred electrons (4), $F$ is the Faraday constant, $C$ is the concentration of OH^−^. According to $Z' = R_{s} + R_{ct} + \sigma\omega^{-1/2}$, it can be concluded that $\sigma$ is the slope of the plot of $Z'$.


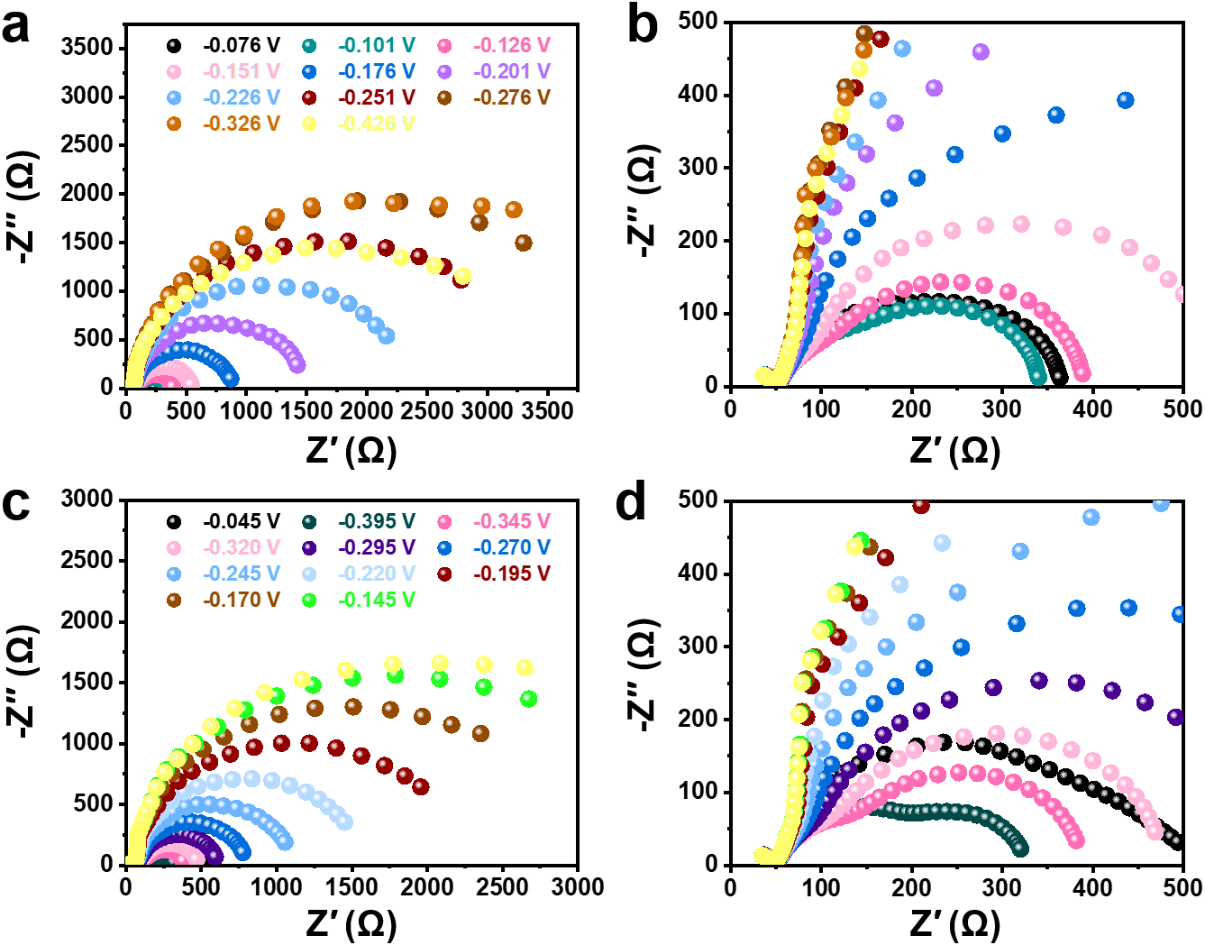


**Supplementary Fig. S16.** Nyquist plots of (a, b) PBMC and (c, d) POMC at various *ΔE* (= *E*_applied_ – *E*_onset_).


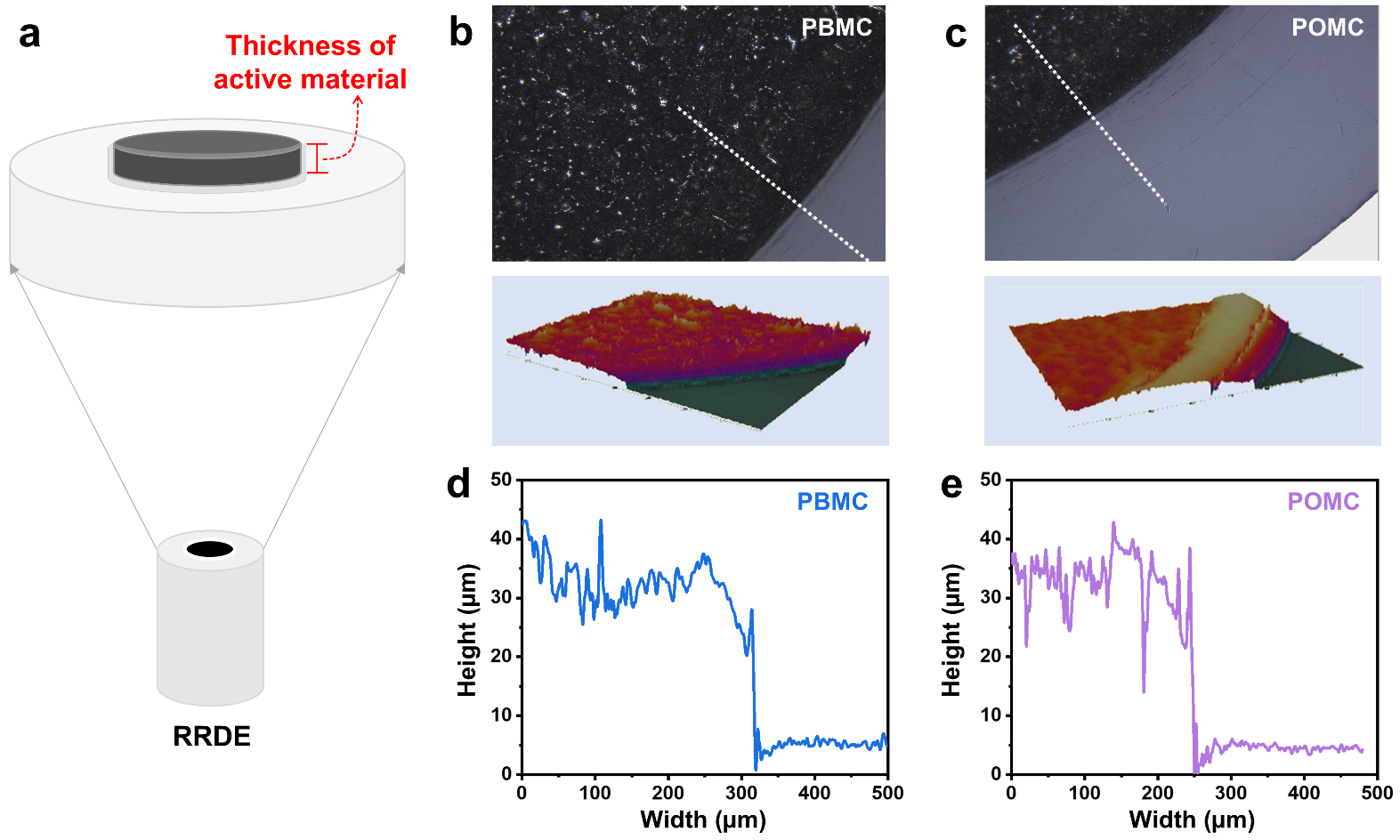


**Supplementary Fig. S17.** (a) Schematic illustration of RRDE and its thickness of active material. Photos and three-dimensional depth view of RDE coated with (d) PBMC and (c) POMC. Stylus profilometry scans along the white dotted-lines in (b) and (c) showing the thickness of (d) PBMC and (e) POMC, respectively, on RDE.


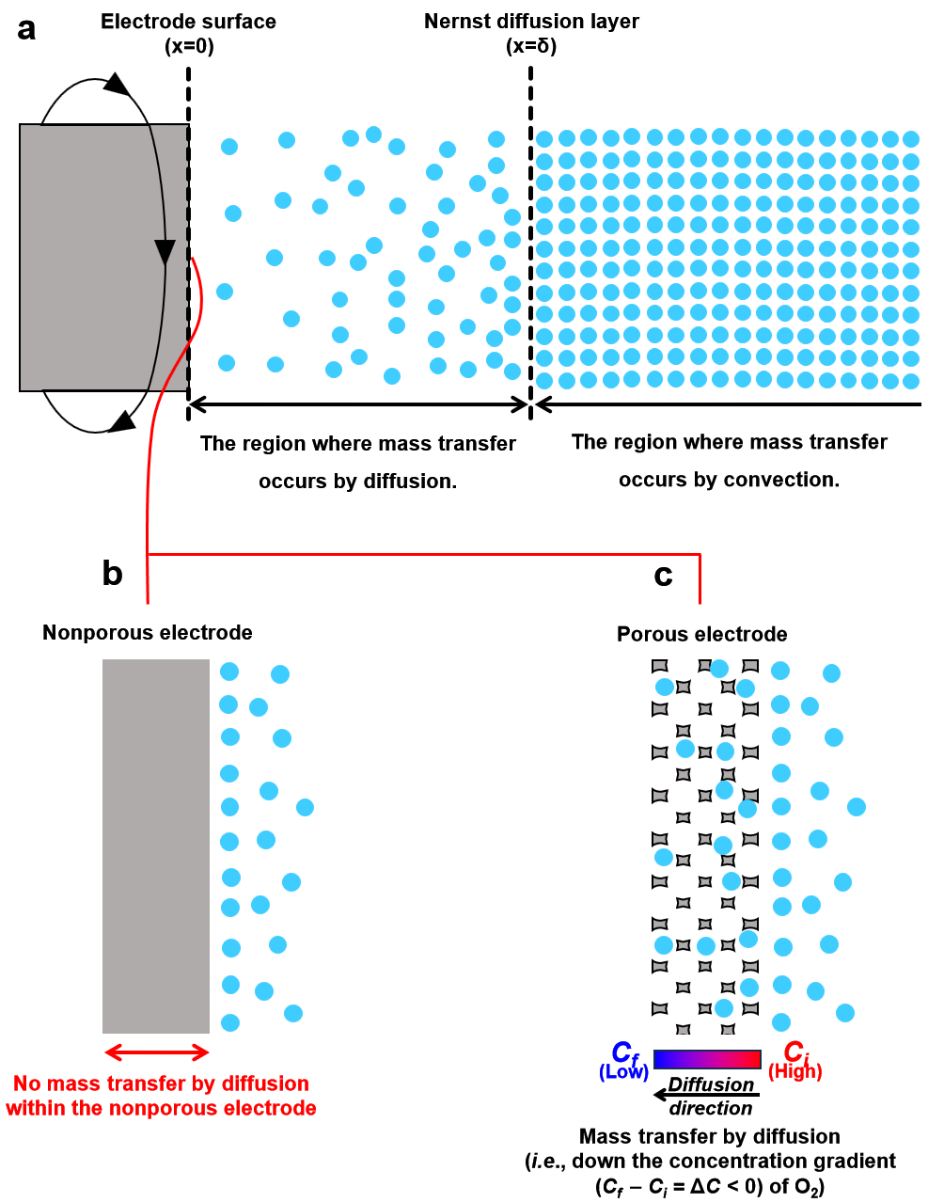


**Supplementary Fig. S18.** (a) Schematic description of mass transfer of reactants between the bulk electrolyte and electrode surface in a forced convection system with rotating disk electrode (RDE). Schematic description of mass transfer by diffusion within the Nernst diffusion layer (NDL) on (b) nonporous, and (c) porous electrode materials.

Supplementary note to Supplementary Fig. S18.

In a forced hydrodynamic condition with the RDE, the mass transfer of reactants takes place by convection and the concentration in the bulk electrolyte is maintained as long as the distance from the electrode surface (x) is below the NDL (*i.e*., x < δ). As the reactants enter the NDL, where the solution is unstirred, the mass transfer is controlled by diffusion as the concentration gradient of the reactants is formed within the layer (*i.e*., 0 ≤ x ≤ δ) (Supplementary Fig. S18a). Based on the Levich equation, which is applicable to the condition that is sorely limited by mass transfer, the thickness of NDL for the RDE can be represented by the following equation:

$$\delta=1.61D^{1/3}\omega^{-1/2}v^{1/6}$$

This indicates that regardless of the type of electrode materials, the thickness of NDL remains unchanged in the diffusion-controlled region if they are exposed to the identical electrochemical system with equal rotating speed.

As the supply of reactants to the surface of electrode occurs at a consistent rate of diffusion by the RDE in the same electrochemical conditions, the rate of mass transfer of reactants to the electrode materials (or active sites) on the electrode surface becomes increasingly important. In the case of nonporous electrode materials (*e.g.,* re-stacked graphene), the mass transfer of reactants to the active sites is highly limited and the steady-state of the RDE is expected to be achieved by only a small limiting current (Supplementary Fig. S18b).

On the contrary, if the nanopores are introduced to the electrode materials, the mass transfer of reactants is expected to increase, but at a varied degree depending on the openness or blockage of the nanopores as described in Supplementary Fig. S18c. Specifically, the formation of nanopores on the surface of nonporous materials generates more free space to facilitate an improved mass transfer of the reactants, therefore, requiring more limiting current to reach steady-state as compared to nonporous materials. In the case of the open porous materials, in which the pores are fully interconnected without any blockage, the mass transfer of reactants occurs much more efficiently, and higher limiting current will be required to achieve the steady-state.


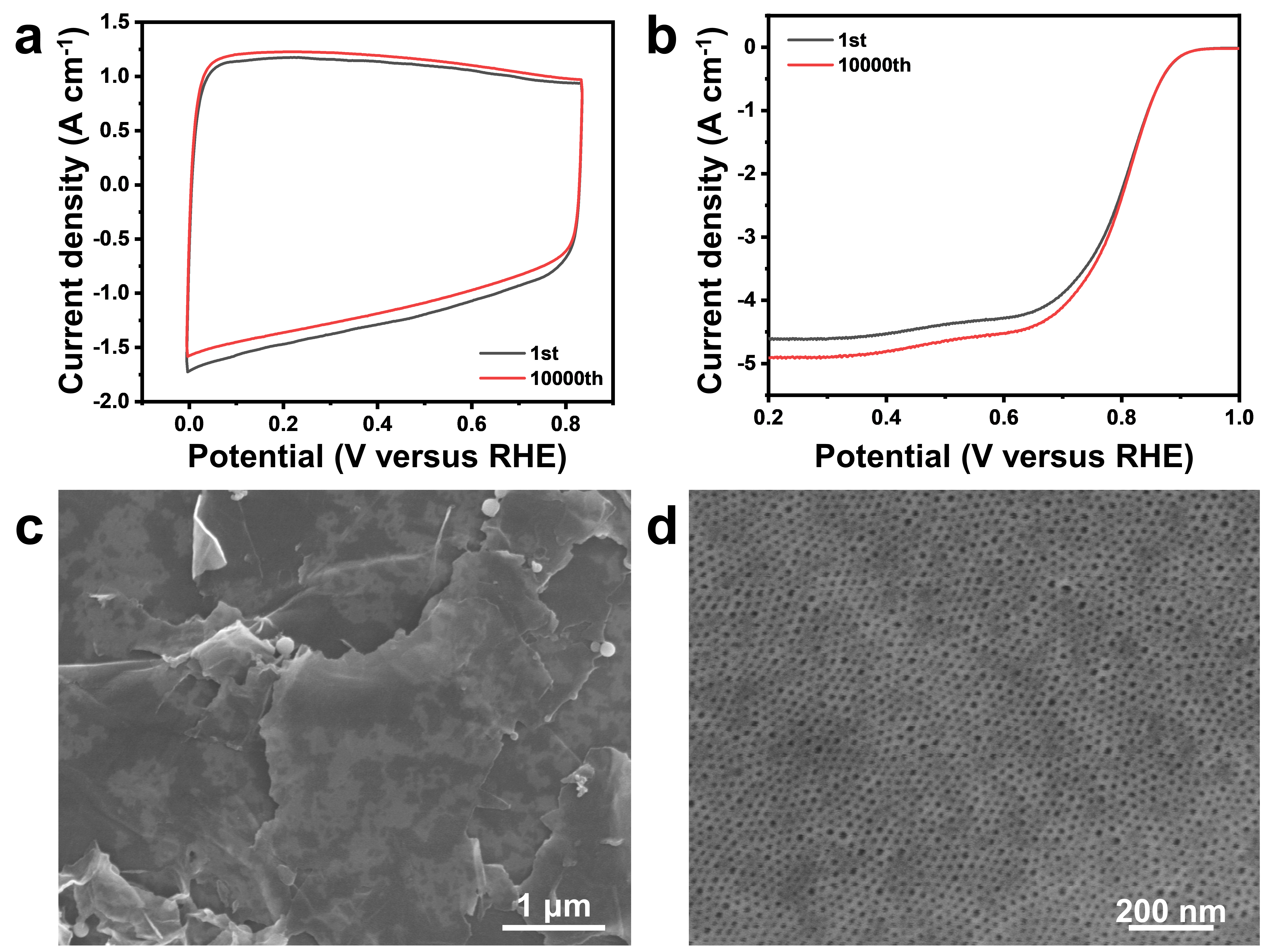


**Supplementary Fig. S19.** (a) 1^st^ (black) and 10000^th^ (red) CV curves at 20 mV s^-1^ of POMC. (b) LSV curves at 10 mV s^-1^ with 1600 rpm of POMC before (black) and after 10000^th^ (red) CV curves. (c, d) SEM images of POMC after 10000^th^ cycle.

**References**

1. Hamada, T., Nara, H., Kim, M., Miyata H., Yamauchi, Y., Organic precursors for tailored synthesis of sulfur- and nitrogen-doped mesoporous carbons: a molecular design approach. *Chem. Commun.* **2024**, 60, 4914–4917.

2. Zhao, Y., Nara, H., Jiang, D., Asahi, T., Osman, S. M., Kim, J., Tang, J., Yamauchi, Y., Open-Mouthed Hollow Carbons: Systematic Studies as Cobalt- and Nitrogen-Doped Carbon Electrocatalysts for Oxygen Reduction Reaction. *Small* **2023**, 19 (48), 2304450.

3. Song, Y., Song, X., Wang, X., Bai, J., Cheng, F., Lin, C., Wang, X., Zhang, H., Sun, J., Zhao, T., Nara, H., Sugahara, Y., Li, X., Yamauchi, Y., Two-Dimensional Metal–Organic Framework Superstructures from Ice-Templated Self-Assembly. *J. Am. Chem. Soc.* **2022**, 144 (38), 17457-17467.

4. Nara, H., Mukoyama, D., Yokoshima, T., Momma, T., Osaka, T., Impedance Analysis with Transmission Line Model for Reaction Distribution in a Pouch Type Lithium-Ion Battery by Using Micro Reference Electrode. *J. Electrochem. Soc.* **2016**, 163 (3), A434.
